# Cellulose-Based Hybrid Hydrogels for Tissue Engineering Applications: A Sustainable Approach

**DOI:** 10.3390/gels11060438

**Published:** 2025-06-06

**Authors:** Elizabeth Vázquez-Rivas, Luis Alberto Desales-Guzmán, Juan Horacio Pacheco-Sánchez, Sofia Guillermina Burillo-Amezcua

**Affiliations:** 1 Centro Universitario de Ciencias Exactas e Ingenierías, Universidad de Guadalajara, Guadalajara 44430, Jalisco, Mexico; 2 División de Estudios de Posgrado e Investigación, Instituto Tecnológico de Toluca, Metepec 52149, Edo. México, Mexico; 3 Instituto de Ciencias Nucleares, Universidad Nacional Autónoma de México, Ciudad de México 04510, Mexico; burillo@nucleares.unam.mx

**Keywords:** cellulose, cellulose derivatives, sustainable resources, hydrogels, stimuli responsive hydrogels, tissue engineering, scaffolds, carboximethyl cellulose, methyl cellulose, hydroxypropylcellulose

## Abstract

Cellulose is a sustainable biopolymer, being renewable and abundant, non-toxic, biodegradable, and easily functionalizable. However, the development of hydrogels for tissue engineering applications presents significant challenges that require interdisciplinary expertise, given the intricate and dynamic nature of the human body. This paper delves into current research focused on creating advanced cellulose-based hydrogels with tailored mechanical, biological, chemical, and surface properties. These hydrogels show promise in healing, regenerating, and even replacing human tissues and organs. The synthesis of these hydrogels employs a range of innovative techniques, including supramolecular chemistry, click chemistry, enzyme-induced crosslinking, ultrasound, photo radiation, high-energy ionizing radiation, 3D printing, and other emerging methods. In the realm of tissue engineering, various types of hydrogels are explored, such as stimuli-responsive, hybrid, injectable, bio-printed, electrospun, self-assembling, self-healing, drug-releasing, biodegradable, and interpenetrating network hydrogels. Moreover, these materials can be further enhanced by incorporating cell growth factors, biological molecules, or by loading them with cells or drugs. Looking ahead, future research aims to engineer and tailor hydrogels to meet specific needs. This includes exploring safer and more sustainable materials and synthesis techniques, identifying less invasive application methods, and translating these studies into practical applications.

## 1. Introduction

Cellulose is the predominant polymer found in plant cell walls and provides structural integrity to plants. However, it can also be sourced from various organisms such as bacteria, algae, and animals, each offering unique advantages regarding the final application and extraction method. Notably, cellulose obtention from plants requires an extensive process to remove other polymers like hemicellulose, lignin, pectin, and other biomass residues, unlike other sources where hydrolysis is the key step [1,2,3,4,5,6,7,8,9,10,11,12].

Natural cellulose exhibits high crystallinity and rigidity, which makes it insoluble in water. Additionally, cellulose has unique properties, such as a highly defined three-dimensional structure, exceptional mechanical strength, and high porosity. In addition, it offers high durability, moldability, chemical and thermochemical stability, biocompatibility, biodegradability, non-toxicity, non-allergenicity, plasticity, selective permeability, and high-water retention ability. Furthermore, the Young’s modulus of bacterial cellulose can reach up to 95 GPa in the lamellar plane due to its exceptional supramolecular structure characterized by strong hydrogen bonds between the fibers.

Crystallinity is a crucial characteristic influencing cellulose’s mechanical, chemical, and physical properties. Seven interconvertible crystalline polymorphs of cellulose have been identified to date, including cellulose Iα, Iβ, II, III_I_, III_II_, and IV_I_, with some studies suggesting the existence of a V or X structure. Natural cellulose primarily exists in the cellulose I crystalline state, featuring eight hydrogen bonds per glucose unit. In contrast, cellulose II has 5.3 hydrogen bonds, making it more chemically reactive. In this regard, integrating natural cellulose into various materials for medical applications presents several challenges due to its crystalline regions. Such regions resist chemical modification due to their well-ordered and robust molecular structure. Consequently, to enhance the reactivity of cellulose, it is essential to modify its structure through chemical or physical processes. Overall, treatments applied to cellulose disrupt the assembly and orientation of the fibrils, resulting in changes to the original arrangement of the crystalline structure, as shown in Table 1.

Currently, cellulose-based hydrogels have great potential for tissue engineering applications. Tissue engineering is an interdisciplinary field focused on restoring or replacing body tissues and organs using biomaterials and bioactive substances to mimic the function of damaged or missing tissue [19]. Several critical characteristics must be carefully considered when designing hydrogels for tissue engineering applications to mimic the natural performance of the extracellular matrix (ECM). One of the primary aspects is surface properties, which play a vital role in regulating cell adhesion and promoting the attachment and proliferation of cells. Porosity is another essential characteristic; proper pore size and interconnection facilitate the efficient transfer and diffusion of nutrients, cells, and other substances while ensuring that the mechanical properties of the hydrogel provide strong support for cell growth and the mechanical demands of the targeted tissue. Another crucial factor is the degradation rate of hydrogel, which must align with the pace of tissue formation to ensure structural integrity during tissue growth and development. Furthermore, the design must effectively distribute proteins and growth factors throughout the hydrogel, creating an environment that fosters cellular diversity and enhances the differentiation of stem cells [20].

In this context, cellulose-based hydrogels exhibit significant characteristics including mechanical strength, biocompatibility, non-toxicity, biodegradability, hydrophobicity, antioxidant, and antibacterial properties. These attributes render cellulose-based materials ideal candidates for tissue engineering applications, as they facilitate the binding of nutrients and growth factors, enable metabolite and gas exchange, and provide spatial guidance for cell growth. Additionally, cellulose-based materials offer stability, hydrophilicity, and numerous chemical modification sites, further enhancing their suitability for tissue engineering applications [21].

Nevertheless, preparing hydrogels for tissue engineering presents significant challenges that require multidisciplinary expertise. The body is a dynamic system, where each tissue and organ possesses distinct characteristics and requirements that conventional hydrogels cannot meet due to their limitations in achieving mechanical, biological, chemical, and surface properties akin to those of the extracellular matrix. Therefore, extensive research has been conducted to modify and customize hydrogels that mimic native ECM functions, tissue, or organs [22].

Recent advancements in this field focus on engineering the structure and properties of hydrogels by manipulating several critical factors, including the chemical composition molecular weight, and concentration of the polymers and other materials involved, as well as the fabrication method, since these factors influence the structure, mechanical characteristics, chemical properties, and surface qualities of hydrogels [23]. Additionally, researchers are exploring innovative techniques to address the significant challenges posed by traditional methods, which often employ toxic crosslinking agents that can be difficult to eliminate once the process is complete. There is also growing concern about using non-biodegradable synthetic materials, and researchers are actively seeking safer, more sustainable, and efficient alternatives, such as cellulose and its derivatives, to enhance hydrogels’ sustainability and functionality [24].

Several techniques, such as enzyme-induced crosslinking, ultrasound, photo radiation, high-energy ionization radiation, click chemistry, and microwaving, are elaborated upon in this paper. Additionally, research studies presented in the Cellulose-Based Hydrogels for Tissue Engineering section illustrate specific methods employed in developing hydrogels designed for the repair, regeneration, or substitution of various tissues or organs within the human body. This paper also discusses techniques for delivering and positioning hydrogel scaffolds in targeted areas, as well as methods for characterizing and assessing the performance of these hydrogels. In this context, the fabrication methods discussed typically involved a blend of techniques, incorporating combinations of cellulose derivatives, cellulose nanofibers, cellulose nanocrystals, and bacterial nanocellulose, along with other materials to create hydrogel matrices and to enhance their structural, chemical, and biological properties. As a result, researchers have reported innovative hydrogels with diverse structures, functionalities, and characteristics, such as stimuli-responsive, hybrid, injectable, bioprinted, electrospun, self-assembling, self-healing, drug-releasing, biodegradable, and interpenetrating network hydrogels, and hydrogels loaded with nutrients, growth factors, or cells, among others. Finally, this paper presents potential vast applications of innovative cellulose-based hydrogels in the field of tissue engineering while also addressing their perspectives and challenges that still need to be addressed, setting the stage for future advancements in tissue engineering applications.

## 2. Cellulose Sustainable Sources and Cellulose Derivatives

### 2.1. Cellulose Structure

Cellulose is the most abundant biopolymer on Earth. It is characterized by a linear polymeric structure of d-glucose units linked by β (1–4) glycosidic bonds. The degree of polymerization, or the number of glucose units in the polymer chain, varies across sources, with some wood sources exhibiting a degree of polymerization of up to twenty thousand units [25,26].

**Figure 1:**
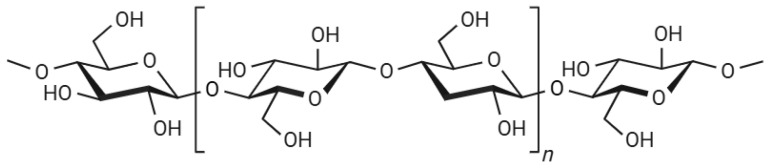
Cellulose network structure.

Each glucose unit (C_6_H_10_O_5_) within the cellulose chain possesses three hydroxyl groups. The spatial arrangement of the β-(1–4)-glycosidic C1–O and O–C4 bonds results in an alternating 180-degree turn of two glucose units along the cellulose chain axis [27]. The parallel stacking of cellulose chains is facilitated by inter- and intra-molecular hydrogen bond networks between their hydroxyl groups, C–H^…^O hydrogen bonds, and van der Waals forces. These interactions lead to well-organized crystalline arrangements, commonly known as crystalline cellulose (Figure 1). Furthermore, increased hydrogen bonding along the chain enhances its stiffness and straightness [26,27].

### 2.2. Cellulose Sources

**Figure 2:**
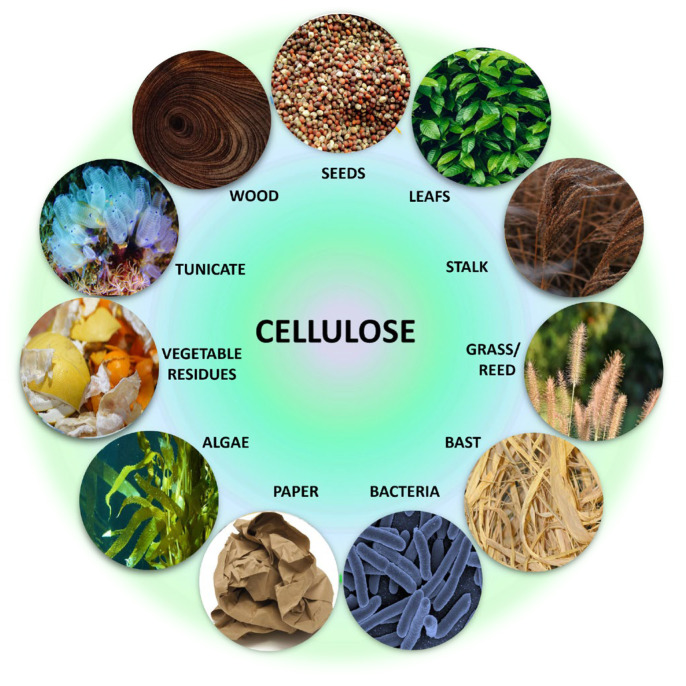
Cellulose resources.

Living organisms synthesize cellulose, typically classified according to its source of origin, as depicted in Figure 2 [28]. Traditionally, cellulose has been obtained from conventional sources such as wood and plants, unlike other sources such as bacteria. However, if not responsibly managed, cellulose production and extraction processes from plants can contribute to deforestation, pollution, and environmental degradation. Additionally, the high demand for wood cellulose and cotton cellulose may surpass the slow growth of trees. Given sustainability goals to conserve natural resources without overexploitation, there is an increasing demand for sustainable alternatives with a positive impact on society and the environment, notably biomass from agricultural waste and bacterial sources, where the degree of cellulose extraction varies among different sources and is also influenced by the extraction method used [29].

#### 2.2.1. Plant Cellulose

Among plant sources of cellulose, cotton fibers offer the advantage of relatively low non-cellulosic components, with approximately 90 wt% purity and 60% crystalline regions, as indicated in Table 2. The second most abundant source of cellulose is wood, which exhibits good mechanical properties and potential antibacterial characteristics due to the presence of natural phytochemicals. Cellulose extracted from wood contains lignin and other polysaccharide compounds, with a cellulose content of 50% by weight. Additionally, cellulose has been extracted from agricultural residues such as oil palm dough waste, jute, banana plants, rice straw [30], wheat straw, corn stalks, cotton stalks, soybean hulls, tomato peels, pineapple leaves, and secondary residues [31]. These sources can be considered good sources of cellulose, and the resulting cellulose can vary depending on the source and the extraction process utilized.

#### 2.2.2. Bacterial Cellulose

Cellulose produced by prokaryotic organisms, known as bacterial cellulose, offers a sustainable alternative to alleviate the demand for plant cellulose. Certain bacterial strains secrete extracellular cellulose primarily as a defense mechanism against various biotic and abiotic stresses. This cellulose aids in enhancing surface adhesion for nutrient absorption, provides protection against UV radiation, fungi, and yeasts, facilitates resource capture and retention, promotes cell cooperation, and even exhibits resistance to antibiotics [43].

Gram-negative bacteria like *Acetobacter*, *Achromobacter*, *Aerobacter*, *Agrobacterium*, *Alcaligenes*, *Azotobacter*, *Escherichia*, *Novacetimonas*, *Rhizobium*, *Pseudomonas*, *Salmonella*, and Gram-positive *Sarcina ventriculi* are among the strains capable of synthesizing cellulose. Some bacteria produce amorphous or paracrystalline cellulose, including Escherichia coli 1094, *Sarcina* strains, and *Agrobacterium tumefaciens*. Others can generate crystalline Iα microfibrils, such as bacteria from the *Komagataeibacter* genus (formerly *Glucanobacter* and *Acetobacter*). These aerobic, immobile organisms can oxidize sugars, aldehydes, and alcohols and convert ethanol into acetic acid in the presence of oxygen [44]. Both *Komagataeibacter* and the *Novacetimonas genus* are tolerant of low pH levels. They are commonly found in decomposing fruits, vegetables, vinegar, fruit juices, and alcoholic beverages, producing significant amounts of bacterial cellulose [45].

Species like *Novacetimonas hansenii* (previously known as *Komagataeibacter hansenii* or *Gluconacetobacter hansenii*), *Komagataeibacter europaeus*, *Komagataeibacter rhaeticus*, *Komagataeibacter medellinensis*, and *Komagataeibacter xylinus* are considered among the most proficient cellulose producers among bacteria. These species have been utilized as model organisms to investigate bacterial cellulose production [46].

**Figure 3:**
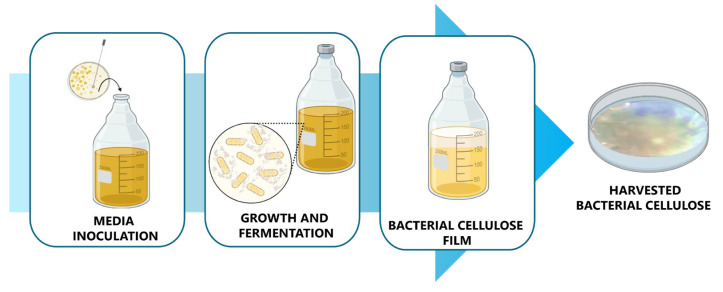
Bacterial cellulose production process.

Bacterial cellulose production involves a fermentation process driven by two primary mechanisms: (a) uridine diphosphoglucose synthesis (UDPGlc) and (b) glucose polymerization by cellulose synthase. Two main types of cultures are utilized for fermentation: static and agitated. In static culture, gelatinous films form on the exposed surface, while in agitated fermentation, irregular pellets are suspended in the liquid medium. Static fermentation offers greater genetic stability (see Figure 3) [47], a higher degree of polymerization and crystallinity, and a higher proportion of cellulose Iα compared to cellulose Iβ in comparison to agitated fermentation [47]. Although agitated fermentation is more straightforward to scale up for industrial production, the emergence of mutant non-cellulose strains could diminish productivity [48].

One significant drawback in bacterial cellulose production is the high cost due to low yield and the need for expensive culture media, which may constitute up to 30% of the total cost. To address this issue, research is underway to identify cost-effective culture media promoting high cellulose yields in short timeframes [49]. In this context, manufacturing bacterial cellulose from agricultural residues or industrial byproducts offers a more sustainable, environmentally friendly, and cost-reducing process. For example, substituting sodium hydrosulfite medium with nicotine-free tobacco residue extract in a two-stage fermentation resulted in a 1.6-fold increase in cellulose yield [50]. The impact of nitrogen sources such as peptone, yeast extract, tea extracts, sodium glutamate, hydrolyzed casein, ammonium sulfate, and glycine on bacterial cellulose production has been investigated. Other studies have shown that undefined crude media can outperform defined media, reducing raw material costs. In this regard, fresh oil palm juice was utilized as an alternative fermentation medium (nitrogen source), achieving a maximum yield of high-quality cellulose [51].

Bacterial and plant cellulose exhibit distinct physical and some chemical properties, despite sharing similar cellulose I and II structures. Bacterial cellulose is extruded through the bacterium’s cell wall, serving as a protective barrier against the environment, while plant cellulose is a structural component. Consequently, bacterial cellulose adopts a gel-like three-dimensional structure, whereas plant cellulose is fibrous. The polymerization degree of bacterial cellulose ranges from 1 to 20 kDa, while in plant cellulose, it falls between 13 and 15.5 kDa [52]. Plant fibers are micrometric, contrasting with the nanoscale dimensions of bacterial cellulose, which typically ranges from 10 to 50 nm in diameter and 100 to 1000 nm in length [49,50,53,54].

Bacterial cellulose derived from static fermentation exhibits a very high level of crystallinity (60–90%), surpassing that of plant cellulose (40%) and cotton fibers (70%). Moreover, its high hydrophilicity enables it to retain up to 1000% of water, unlike the 60% retained by plant cellulose [55]. These properties vary depending on the strain and growth media composition, with strain type determining the crystalline structure and growth media influencing molecular dimensions [56]. Generally, bacterial cellulose is chemically purer as it lacks hemicellulose, lignin, and other components. Additionally, its malleability enables it to grow into almost any desired shape.

In the biomedical field, bacterial cellulose has been found to be an ideal wound dressing due to its ability to accelerate healing, prevent microbial infections, and restore skin structure and function. It enhances the sorption of serous drainage and promotes air permeability, thus safeguarding the formed skin tissue. Advances have also been made in tissue engineering by mimicking bacterial cellulose nanofibers to collagen. Bacterial cellulose has been investigated for various applications in tissue engineering, including artificial skin, cartilage replacement tissue, artificial blood vessels, corneal stroma tissue, dental implants, and as a pharmaceutical excipient in transdermal and oral drug delivery systems, among others [57,58].

#### 2.2.3. Algal Cellulose

The term “algae” encompasses a diverse group of photosynthetic organisms that are conventionally divided into two categories. First, there are macrophytes, which are multicellular marine organisms commonly referred to as macroalgae. Second, there are unicellular or colonial microalgae, which may consist of either Prokaryotes (such as Cyanobacteria, also known as blue-green algae) or Eukaryotes inhabiting various water bodies or land [59].

Macroalgae are classified based on their morphological and anatomical characteristics, although a universally accepted classification system has yet to be established. The commonly used classification system roughly categorizes algae based on their pigmentation. *Chlorophyta* comprises green algae, which produce chlorophyll. The *Rhodophyta* families are red algae, characterized by their predominant phycobiliprotein content. Lastly, the *Ochrophyta* group includes brown algae, formerly known as *Phaeophyta*, distinguished by their high concentration of fucoxanthin, which lends them their brown coloration. The growing interest in utilizing sustainable and natural materials has contributed to the increasing market demand for seaweed. Seaweed cultivation offers several advantages, requiring no land, fertilizers, pesticides, or freshwater. Moreover, seaweed exhibits a faster growth rate than terrestrial plants [60].

Various taxonomic groups of algae possess cellulose, hemicelluloses, glucoproteins, and specific polysaccharides in their extracellular walls. This includes green algae such as *Chlorophyta* and *Charophyta*, red algae like *Rhodophyta*, brown algae belonging to the class Phaeophyceae, and microalgae such as *Ochrophyta*. However, the composition can vary among species. In particular species like *Dinophyta* (armored dinoflagellates), there may be a complex extracellular covering called an epitheca, which might also feature a cellulose plate armor known as the teca. Polysaccharides in the cell wall include pectins, ulvans, agars, carrageenans, or alginates, some of which need to be removed during cellulose extraction, especially in green algae [61]. Furthermore, the cellulose content has been observed to differ among various algae groups and even among individual organisms. However, reported cellulose content for dried algae can range from 0.85% to 34% [60].

The extraction of cellulose from algae, whether in the form of nanofibrils or nanocrystals, involves various purification steps. One of the initial steps is biomass decomposition and cellulose separation, followed by pretreatment of the cellulose pulp through mechanical, biological, or chemical means to facilitate fiber delamination and mechanical fiber disintegration. Nanocrystals are typically obtained through strong acid hydrolysis of purified cellulose pulp with sulfuric acid (60%). Cellulose nanocrystals measure between 2 and 15 nm wide and between 100 and 500 nm long, while cellulose nanofibers measure between 20 and 50 nm wide and between 500 and 1500 nm long [61]. Algal cellulose has also been applied as reinforcement in rigid polyurethane foam [62], in polymeric nanocomposites, as a filtering medium [63], as a biologically based flocculant [64], for paper production, cellulosic ethanol, bioplastics [65], and many other applications in biomedical, construction, food, environmental, and industrial fields.

#### 2.2.4. Animal Cellulose

Tunicates, also known as urochordates, are marine invertebrate animals belonging to the diverse subphylum of *Chordata*. There are three classes of tunicates: *Ascidiacea*, *Thaliacea*, and *Appendicularia*, comprising over 2000 species of ascidians, about 72 species of thaliaceans, and approximately 20 species of appendicularians. Tunicates are enveloped in a tunic or cellulosic extracellular matrix of highly crystalline cellulose nanofibers synthesized by a cellulose synthase [66,67].

Tunicate cellulose exhibits a unique crystalline structure, primarily composed of type Iβ cellulose, characterized by a monoclinic cell with two chains, resulting in high density and providing chemical and thermodynamic stability. Zhao et al. [68] reported the extraction of tunicate cellulose using acid hydrolysis, yielding ribbon-like, nearly pure Iβ cellulose nanocrystals approximately 8 nm in height, 20 nm in width, with lengths ranging from 100 to 4000 nm and 85–100% crystallinity. Additionally, tunicate cellulose nanofibrils typically have lengths ranging from 100 nm to several micrometers (2 μm) and widths ranging from 10 to 30 nm, exhibiting a tensile modulus of 143 GPa and a length–diameter ratio of 70–100, which surpasses cellulose from plant sources such as cotton or wood (aspect ratio: 10–15) [69].

Tunicate biomass contains low levels of different components, facilitating cellulose extraction without severe chemical treatment and producing high yields. Moreover, tunicates grow faster than typical terrestrial cellulosic sources, making them a potential eco-friendly alternative for cellulose production that can reduce land requirements and aid in carbon sequestration back to the ocean floor [69,70].

Tunicate nanocellulose is typically isolated via acid hydrolysis to remove amorphous regions. This process also results in surface functionalization, producing long and highly crystalline whiskers capable of forming stable suspensions [71]. Nanocellulose isolated from the ascidian *Styela clava* was dispersed in organic solvents, facilitating the formulation of nanocomposites with reinforced mechanical properties. In biomedical applications, it exhibited osteoconductive effects on injured bone, and when applied in liquid form or as a membrane, it accelerated wound healing [71,72].

The applications of tunicate cellulose are expanding as this material is still considered relatively underdeveloped. Due to its high crystallinity and strength, it can be utilized to fabricate various types of materials. Wu et al. [73] prepared aerogels that exhibited stability after immersion in pH solutions (0–12) for 72 h. These aerogels had a contact angle above 150° and demonstrated the ability to separate oil and water in more than 10 cycles for different water–oil mixtures, achieving 90% separation efficiency. In the biomedical field, tunicate cellulose nanofibrils have been combined with living cells for cartilage and soft-tissue repair, as they possess dimensions similar to collagen. Industrial production of these tissue types seems feasible and cost-efficient [74]. Other applications include edible films [75], enteric capsules [76], membranes and weavable sensing fibers [77], mechanothermal chromic hydrogels [78], and self-healing, high-strength rubber [79].

#### 2.2.5. Cellulose Derivatives

**Figure 4:**
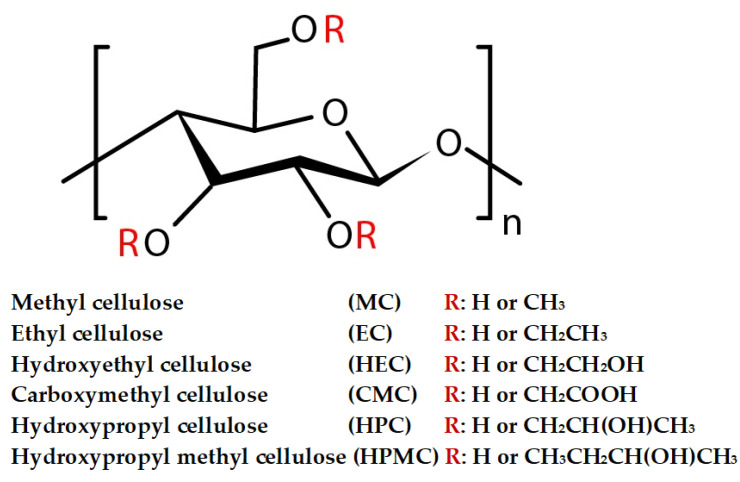
Chemical structure of the most common cellulose derivatives.

Cellulose derivatives are chemical modifications aimed at introducing various functional groups or chemical substituents to enhance, alter, or improve cellulose properties, thus paving the way for developing new functionalities. Chemically, cellulose undergoes modification by substituting its three available hydroxyl groups in each anhydroglucose unit with functional groups like acids, chlorides, and oxides [80]. This involves reactions such as esterification, etherification, and substitution, leading to a diverse range of derivatives, including esters of organic and inorganic acids and ionic and non-ionic ethers. Some derivatives feature combined functional groups within the same chemical category, such as acetate/butyrate and acetate/propionate, or across distinct classes like ether and ester, as seen in carboxymethyl cellulose acetate butyrate, as shown in Figure 4. Additionally, derivatization can sometimes serve as an intermediate product [81].

The derivatization of cellulose can occur through either homogeneous or heterogeneous reactions. In homogeneous reactions, cellulose remains in a single phase throughout the process, typically achieved by dissolving cellulose beforehand in either a derivatizing or non-derivatizing solvent, thereby transforming it into a derivative. Conversely, reactions are heterogeneous if at least two phases remain unchanged [81]. Under heterogeneous conditions, the reactivity of the hydroxyl groups in the anhydroglucose unit can be influenced by inherent chemical reactivity, steric effects from the reacting agent, or steric effects due to the supramolecular structure of cellulose. Generally, the hydroxyl groups at the C2 and C3 positions behave as secondary alcohols, while the hydroxyl group at C6 acts as a primary alcohol [82]. Cellulose derivatization follows three primary routes of reaction: esterification, etherification, and substitution reactions, with the choice of reaction type contingent on the desired cellulose derivative and its intended application.

## 3. Synthesis of Cellulose-Based Hydrogels

Hydrogels are three-dimensional networks formed by crosslinked polymeric chains that exhibit hydrophilic properties. These structures can undergo reversible swelling and deswelling by absorbing or desorbing water; They can absorb and retain substantial amounts of water or aqueous solutions, including physiological fluids, with capacities ranging from ten to thousands of times their weight in a relatively short timeframe. Furthermore, hydrogels can sustain a saturated liquid state even under pressure for extended periods without dissolving in water. During the swelling process, initial water molecules (bound water) are quickly drawn into the hydrogel network by the polar groups in the polymer chain. The absorption of additional water molecules follows this due to the osmotic pressure of the interstitial water and free water [83].

**Figure 5:**
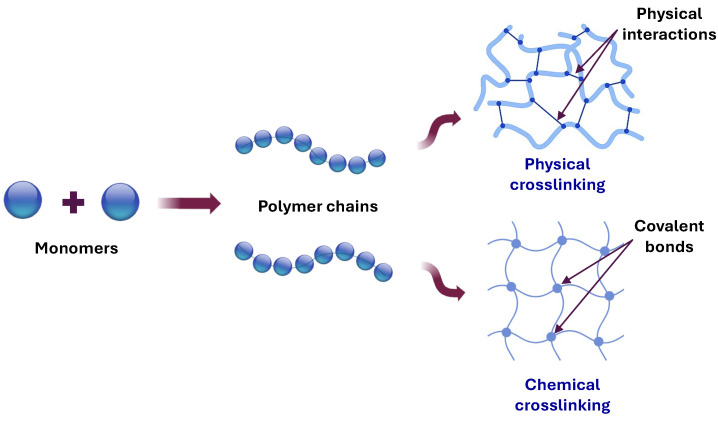
Hydrogel physical and chemical crosslinking methods.

Since cellulose is a polymer rich in hydroxyl groups that cannot form hydrogels on its own, it can be used as a component of hydrogels when combined with other polymers or through various chemical modifications and functionalization. Three-dimensional, highly crosslinked hydrophilic cellulose hydrogels can be formed through weak hydrogen bonds, ionic and van der Waals interactions, or covalent bonds, which offer structural support to the polymers in their intact form. In this regard, cellulosic hydrogels can be developed by integrating natural or synthetic polymers using physical or chemical methods as presented in Figure 5.

Cellulose-based hybrid hydrogels can be produced using various types of polymers. Synthetic versions are commonly obtained by copolymerization with polymers such as poly(vinyl acetate), poly(acrylic acid), poly(ethylene glycol), and poly(N-isopropylacrylamide). However, these synthetic hydrogels may have disadvantages, including poor biodegradability, high costs, and the potential to generate toxic degradation products [84]. In contrast, natural polymers, such as pullulan, dextran, starch, chitosan, chitin, agarose, alginic acid, alginate, hyaluronic acid, chondroitin sulfate derivatives, heparin, pectin, polypeptides, proteins, gelatin, collagen, albumin, fibrin, fibrinogen, silk, genetically engineered proteins, as well as DNA and RNA, can be employed effectively [85].

Producing cellulosic hydrogels through physical and chemical crosslinking strategies allows adjustments to properties for various applications by modifying the proportions of hydrophilic and hydrophobic components or incorporating active recognition motifs. This results in hydrogels with specific characteristics that can overcome the challenges faced by traditional hydrogels, such as heterogeneous structures and network defects, thereby enhancing mechanical properties and improving the diffusion rates and activity of biologically active molecules [86].

### 3.1. Physically Crosslinked Hydrogels

Physically crosslinked hydrogels, often reversible or pseudo-hydrogels, are synthesized through non-covalent interactions, including electrostatic attractions and hydrophobic forces. These mechanisms lead to the establishment of various types of bonds, including ionic, hydrogen, hydrophobic, and both inter- and intramolecular bonds. The unique properties exhibited by these hydrogels stem from their ability to undergo reversible changes in their structure, making them desirable for a wide range of applications. Changes in physical conditions, such as ionic strength, temperature, and pH, can significantly alter the structure of these polymer networks. Although they may exhibit poorer mechanical properties and chemical stability than hydrogels formed by covalent bonds, physically crosslinked hydrogels are noted to be similar to biological systems due to their assembly dynamics, making them valuable for tissue engineering [87,88].

#### 3.1.1. Crosslinking by Ionic Interactions

Hydrogels can be effectively synthesized due to the ionic characteristics of the functional groups within the leading polymer chains. The process commonly involves in situ crosslinking, which facilitates the transformation from a polyelectrolyte solution to a gel state (sol–gel) by introducing multivalent counterions. The resultant material is classified as polyion or polyelectrolyte and coacervate complexes. Various factors, including pH, ionic strength, the nature of the counterion, and the functional charge density of the solution, influence gel formation. These parameters are critical in determining the properties and applications of the resulting hydrogels [89].

In this regard, obtaining a metallo-hydrogel with multi-responsive reversible fluorescent properties was possible through a dual cationic crosslinking strategy that combined interpenetrating networks and an ionic crosslinking–rehydration process. Metal cations (Ca^2+^) were introduced consecutively into the alginate–carboxymethyl–chitosan permeant matrix to enhance the fluorescence intensity. The second loading of metal ions was added to tune the hydrogel’s properties, such as mechanical strength, shape memory behavior, and reversible responsiveness to pH/cation-dependent fluorescent properties. The evaluation results showed that the hydrogels presented a tunable fluorescent emission in response to cations and pH conditions, which activated and deactivated the fluorescent response when exposed to ethylenediaminetetraacetic acid with Mn^+^ [90].

#### 3.1.2. Hydrogels Crosslinked by Hydrogen Bonds

The most widely recognized physical interactions in supramolecular chemistry are hydrogen bonds, also known as H-bonds. Due to their versatility and direction, these bonds are essential for the structural integrity of many biological molecules and biological processes, including molecular recognition, DNA replication, and protein folding [91]. Hydrogen bonding is an electrostatic interaction between an electron-deficient hydrogen atom (donor) and an electronegative atom with accessible nonbonded lone pairs of electrons, such as N, O, or F (acceptor). Different factors, such as the solvent type, the amount, and the arrangement of H-bond donors and acceptors, define the strength of hydrogen bonds. When designing supramolecular hydrogels, H-bonds are preferred due to their high strength, orientation, and dynamic reversibility, in contrast to other non-covalent bonds. H-bonds can range from highly dynamic to quasi-covalent, providing materials with high tunability and flexibility. However, despite all these properties, hydrogen bonds are likely to break easily under high shear forces [91,92].

In this context, ion-conducting hydrogels with electrical conductivity, good mechanical properties, and improved antifreeze capabilities have been synthesized in a single preparation process through hydrogen bonding interactions. Wang et al. [93] used a sodium carboxymethyl cellulose/PVA solution and cellulose nanocrystals as reinforcement. The hydrogel obtained remained flexible and conductive, with a conductivity of 0.021 S/cm at room temperature and 0.014 S/cm at 70 °C. Regarding its mechanical properties, it exhibited around 1.4 MPa for uniaxial tension and around 1018% for deformation, in addition to presenting resistance to freezing and transparency. In another study, carboxymethylcellulose hydrogels were obtained by re-soaking after freezing and thawing. FTIR results suggested that the carboxyl groups of the carboxymethylcellulose were protonated during soaking in an acid solution, thus facilitating hydrogen bonds between the polymer chains. Furthermore, the carboxymethylcellulose hydrogels suffered strain hardening due to increased Young’s modulus and higher strain levels. Also, sub-failure tests showed high recoverability and electrical conductivity, making them suitable for applications in wearable electronic devices and rugged sensors [94].

#### 3.1.3. Freeze–Thawing Method

**Figure 6:**
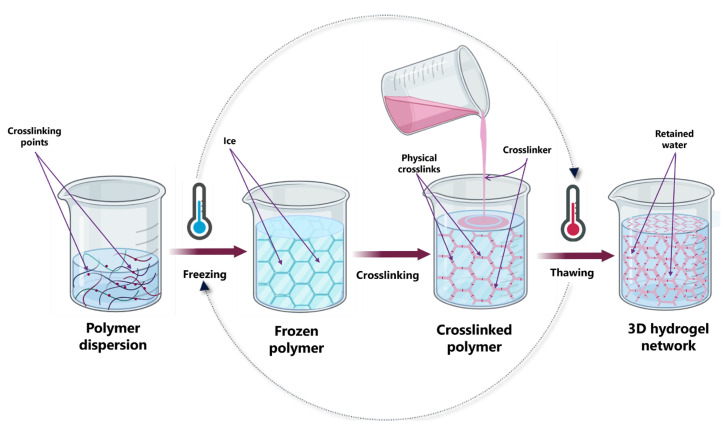
Freeze–thaw method consists of repeatedly freezing and thawing a polymer solution to induce phase separation and create porous structures.

Freeze–thaw crosslinking is a physical method used to form hydrogels (see Figure 6) which operates without crosslinking agents, relying on the establishment of intermolecular interactions, specifically hydrogen bonds, electrostatic forces, and hydrophobic interactions, among polymer chains. The polymer network must meet two critical criteria for effective hydrogel formation via this technique: 1. There must be sufficient intermolecular solid interactions between polymer chains to establish a robust and stable network. 2. The polymer network must allow for adequate water absorption and retention to maintain the hydrogel properties. This process typically involves crystallizing low molecular weight solvents or solutes through a freezing cycle to decrease the spatial distance between polymer chains, thereby enhancing polymer concentration. As a result, polymer chains can align and associate through covalent and hydrogen bonding, ultimately forming an interconnected network [95].

Using this methodology, Pavandi et al. [96] prepared carboxymethyl cellulose and polyvinyl alcohol (CMC/PVA) hydrogels using a freeze–thaw method. The polymer solutions were frozen for five hours, repeating this process five times until a hydrogel displaying the highest swelling ratio or gel fraction was obtained. Ion adsorption studies showed that the hydrogels obtained could adsorb copper and zinc cations, obeying zero-order kinetics.

#### 3.1.4. Crosslinking by Host–Guest Interactions

Another hydrogel preparation technique, usually called complementary, ligand-receptor, lock-and-key, or host–guest interaction, consists of creating complexes between two or more host or guest molecules or ions by noncovalent binding forces and selective interactions. Generally, a molecule with a large cavity volume serves as the host, while a guest molecule typically has a comparable shape and interacts with the host to provide selectivity between the two of them, a phenomenon known as molecular recognition. A combination of several noncovalent interactions, including hydrogen bonding, π–π stacking, electrostatics, hydrophobic interactions, metal–ligand coordination, and van der Waals, may occur between two corresponding compounds. These interactions are reversible and allow the design of structures for processes that imply repeated assembly and disassembly by highly selective recognition of a specific target molecule. Host monomers exhibit significantly stronger complexation and enhanced targeted recognition capabilities than guest monomers. Prominent host molecules such as cucurbiturils, cyclodextrins, and crown ethers are instrumental in constructing various supramolecular polymers characterized by distinct macromolecular architectures. Furthermore, these host molecules can be readily modified, allowing for versatility in their corresponding polymer chains, which enhances their functionality in diverse applications. Nevertheless, although host–guest supramolecular polymers offer many advantages from their specific hydrophilic and hydrophobic structure, only a few host–guest units have been used to produce cellulose-based hydrogels owing to their challenging process. Cellulose host–guest hydrogels can be designed with self-healing, stability, and adhesion properties [91,97,98].

For instance, a spherical double network-structured hydrogel containing cyclodextrins able to encapsulate hydrophobic substances was prepared by electrostatic interactions between a positively charged core of hydroxyethyl cellulose-modified chitosan and a negative sulfo-β-butylether-cyclodextrin (SEB-β-CD) host molecule. The synthesized hydrogel beads demonstrated a remarkable ability to encapsulate a hydrophobic guest substance within a mere 7 min. Furthermore, these hydrogels exhibited a consistent release profile of the guest substance when placed in a buffer solution. This release mechanism was effectively regulated through ion exchange processes involving SEB-β-CD, highlighting the potential for controlled delivery applications in various fields [99]. In the same line of investigation, Jiang et al. [100] worked on the synthesis of an injectable hydrogel to precisely administer a drug. The study employed a modular preassembly process, focusing firstly on the synthesis of several key compounds: adipic dihydrazide-grafted carboxyethyl cellulose, ethyl-1-adamantane 4-formyl benzoate, β-cyclodextrin-grafted carboxyethyl cellulose, and β-cyclodextrin-grafted 4-formyl benzoate carboxyethyl cellulose. Later, these synthesized materials underwent crosslinking through a series of freeze–thawing cycles, enhancing their structural integrity and potentially their functional properties. This approach is indicative of advances in material science, particularly in the development of novel biopolymers with tailored functionalities. Additionally, hydrogels that incorporated doxorubicin hydrochloride revealed impressive self-healing properties, injectability, and rapid recovery in physiological conditions without external stimuli. Additionally, hydrogels with dynamic acyl hydrazone linkages displayed hydrolytic degradation after 30 days and demonstrated pH-responsive behavior. In vivo application tests further confirmed that the engineered hydrogel was able to effectively deliver doxorubicin hydrochloride, enhancing its antitumor efficacy while reducing associated side effects.

### 3.2. Chemical Crosslinking

In contrast with physical crosslinking, chemical crosslinking produces covalent bonds between polymer chains. Therefore, hydrogels keep their structure permanently and are more stable. Chemical crosslinking can be achieved via chemical or radical reactions. Chemical reactions require the presence of monomers, an initiator, and a crosslinking agent. Crosslinking agents mediate bonding between functional groups such as -COOH, -NH_2_, -OH, -CONH_2_, -CONH, and -SO_3_H [101]. At the same time, radical reactions are achieved by irradiating polymeric solutions to produce free radicals that link through covalent bonds, forming insoluble hydrogels [102].

#### 3.2.1. Click Chemistry Reactions

Click chemistry is widely used in hydrogel synthesis due to its fast reaction rate, high selectivity, simple reaction conditions, and minimal byproducts. It refers to a group of cycloaddition reactions with similar characteristics, nucleophilic ring-opening reactions, carbonyl chemistry of the nonaldol type, and addition to carbon–carbon multiple bonds. These reactions feature simple conditions, preferably employ either no solvent or a benign and easily removable solvent such as water and allow for straightforward product isolation. If purification is necessary, it must be achievable through nonchromatographic methods such as crystallization or distillation. Generally, click reactions can be classified into four different types: the Alkyne–Azide Cycloaddition Reaction, the Strain-Promoted Azide–Alkyne Cycloaddition (SPAAC) Reaction, the Thiol–Ene Reaction, and the Diels–Alder (DA) Reaction [103,104,105,106,107,108,109,110,111,112,113,114].

(a) Alkyne–Azide Cycloaddition Reaction: A reaction between azide groups and alkynes leading to the formation of 1,4-disubstituted and 1,5-disubstituted 1,2,3-triazole rings. This process requires elevated temperatures, and when catalyzed by copper ions, it exclusively produces the 1,4-disubstituted isomer. This specific isomer exhibits resistance to oxidation under acidic conditions, is chemically inert to hydrolysis, and has the ability to form hydrogen bonds. This technique has proven useful for synthesizing hydrogels with key advantages, including rapid gelation and high product yields, and the copper-catalyzed version ranks as the most frequently utilized among click reactions. However, copper catalysis is not favorable for applications in tissue engineering due to potential cytotoxicity risks. In this context, Okulmus et al. [104] synthesized multicomponent hydrogels by crosslinking bacterial cellulose (BC), hydroxypropyl methylcellulose (HPMC), and hyaluronic acid (HA) through the Azide–Alkyne Cycloaddition Reaction, catalyzed by copper, at ambient conditions for 24 h. First, hyaluronic acid was prepared using 1-azido-2,3-epoxypropane, and alkyne-terminated cellulose was also ready for the synthesis of the multicomponent hydrogel. The resulting hydrogels proved to be suitable for wound dressing applications. An in vitro cell culturing MTT assay demonstrated that the hydrogels were able to promote cell proliferation, adhesion, and spreading of 3T3 cells.
(b) Strain-Promoted Azide−Alkyne Cycloaddition (SPAAC) Reaction: This reaction is performed at room temperature with no catalyst, between cyclooctyne derivatives and azides, producing aromatic triazoles. Cyclooctyne is the smallest, isolatable, remarkably stable cyclic alkyne whose structure affects the reaction kinetics. Therefore, its reactivity can be improved by introducing electron-withdrawing groups such as fluorine or sp^2^-hybridized atoms into its ring structure. Additionally, cyclooctane derivatives, including bicyclononynes and difluorinated cyclooctyne (DIFO), can be obtained by fusing cyclopropane units. These non-metal-catalyzed reactions facilitate the development of tailored injectable hydrogels and microstructured gels by carefully controlling factors such as space and time. In a study conducted by Nouri-Felekori et al. [105], azide and alkyne moieties were introduced into the structure of citric acid-modified hydroxyethyl cellulose. Through a strain-promoted azide–alkyne cycloaddition, also known as bioorthogonal click chemistry, a hydrogel was formed. Characterization of the hydrogel showed a porous interconnected microarchitecture adequate for cartilage tissue application. Also, the swelling degree reached about 650%, and the mechanical characteristics of the sample were comparable to those of natural cartilage tissue. In vitro biological assays proved that the hydrogel had significant biocompatibility, chondrogenic ability, and bioorthogonal features.
(c) Thiol–Ene Reaction: Reactions between thiols and various functional groups are common. Common reactions between thiol groups and alkenes are carried out under light exposure or thermal initiators to form thioethers. The reaction is highly selective and can be carried out in water, with a yield close to 100%. This technique allows the alteration of the internal spacing of hydrogels by controlling the time, place, speed, and light exposure of the reaction. Moreover, adverse effects caused by ultraviolet light initiation can be avoided by regulating the wavelength and the dose [106,107,108,109,110].

Atmani et al. [106] reported a responsive hydrogel formation via a modular one-pot approach to produce cellulose- and xylan-based allyl-functionalized polysaccharide arbamates, using polysaccharide phenyl carbonates as activated compounds. By fine-tuning the degree of substitution of functional groups, it was possible to control and enhance the gelation and printability properties. Hydrogels were obtained by rapid gelation induced through UV irradiation at 365 nm without the use of harmful catalysts. The produced hydrogels displayed high porosity and interconnectedness, mechanical resistance, and swelling ratio. Tests showed pH-dependent gelation behavior, with optimal results under neutral or acidic conditions. Three-dimensional printing trials demonstrated the material’s rapid shaping capabilities.

In another work, amphiphilic hydrogels were produced by poly(urethane) click chemistry and carbodiimide-mediated green functionalization procedures. The polymers were synthesized to maximize photo-sensitive group grafting while preserving their functionality and were then utilized to prepare thermo- and visible-light-responsive thiol–ene photo-click hydrogels. Green light-induced photo-curing enabled the formation of a more developed gel state with improved resistance to deformation, and the addition of triethanolamine as a co-initiator enhanced the photo-click reaction [108].

In another instance, a cellulose–keratin polymer was obtained by a “thiol–ene” click reaction. Firstly, the allyl cellulose was prepared in an alkali–urea system and then reacted with keratin under UV irradiation. The resulting gel, formed by casting, was easily twisted, bent, and folded, exhibiting excellent water absorption properties. Additionally, it could be functionalized with fluorescence, thermochromicity, and flame retardancy [109]. Troncoso-Alfonso et al. [110] reported a series of plasmonic hydrogels that were orthogonally photo-crosslinked via thiol–ene click chemistry. Using hydrogel-forming polymers such as gelatin, alginate, and carboxymethylcellulose modified with complementary thiol and norbornene groups, the hydrogels were tailored to have specific chemical backbones. These hydrogels met the biocompatibility and printability requirements to be used as 3D cellular scaffolds and showed potential for real-time and in situ detection of biorelevant metabolites.

(d) Diels−Alder (DA) Reaction: The Diels–Alder (DA) reaction is a cycloaddition process that involves an electron-rich diene and an electron-deficient dienophile to create a six-membered ring. This reaction is known for its high selectivity and producing no byproducts; it occurs most rapidly in the presence of water and can be performed with no coupling agent or catalyst. The maleimide–furan reaction, in particular, is extensively utilized for producing hydrogels, which are essential in tissue regeneration and cell encapsulation applications [111,112]. As an illustration, a Diels–Alder reaction was utilized to create hydrogels based on hydroxypropyl methylcellulose. The initial phase consisted of altering hydroxypropylmethylcellulose (HPMC) with a diene compound containing carboxyl groups, which was produced from the synthesis of furfurylamine and succinic anhydride. Following this, dienophile groups were incorporated into HPMC through a coupling reaction with N-maleoyl alanine, employing N,N′-dicyclohexylcarbodiimide and 4-dimethylaminopyridine. Next, the furan- and maleimide-modified HPMC were dissolved in water, leading to gelation at a specified temperature after a certain duration. The duration for gelation was reduced when changes were made to the temperature and concentration of the solution, and the presence of water influenced the kinetics of the Diels–Alder reaction. The swelling characteristics showed that the swelling ratio rose with an increase in temperature [112]. In another study, toluene diisocyanate was employed as a spacer to graft Diels–Alder moieties, such as furyl and protected maleimido moieties, onto cellulose nanocrystals. The reaction time and molar ratio of reactants positively influenced the grafting efficiency. Further characterization confirmed that the grafted moieties and cellulose nanocrystals remained intact after the reaction. However, side reactions were also observed, which impacted the click chemistry reaction on cellulose nanocrystals [113].

#### 3.2.2. Pseudo Click Chemistry Reactions

Reactions involving specific functional groups that exhibit complementary properties and can create junctions between their chains may seem similar to click reactions. For instance, the formation of Schiff bases by interactions between amino carboxylic acids and isocyanates with hydroxyl or amine groups occurs. Additionally, Michael Addition reactions can establish stable network structures through covalent bonds between polymer chains.

(a) Schiff Base Reactions: These are condensation reactions that involve the nucleophilic attack on electrophilic carbonyl groups of aldehydes or ketones, forming Schiff bases. Hydrogels have been developed based on imines and their derivatives, such as hydrazones and oximes, which are the products of reactions between aldehydes or ketones (i.e., glutaraldehyde or dialdehydes) with primary amines, hydrazides, and aminooxy groups, respectively. Additionally, benzoic Schiff base linkages, including benzoic imines, hydrazides, and oximes, are generated by connecting benzoic aldehydes with amines, hydrazides, and aminooxy groups, correspondingly. Hydrogels developed in situ maintain stability under physiological conditions. In this context, hydrazones and oximes exhibit greater intrinsic stability than imines, while acylhydrazones present improved hydrolytic stability compared to both hydrazones and oximes [12,114,115,116].

Even in moderate circumstances, Schiff bases can react reversibly, enabling hydrogels with the capacity for self-healing to restore their structures and functions following damage. In addition, reactions involving Schiff bases are characterized by their brevity, high chemical selectivity, and the absence of toxic byproducts, making them efficient and environmentally friendly processes [114,115,116]. Hydrogels produced through this method are characterized by their biocompatibility, injectability, self-healing properties, and responsiveness to pH changes. These qualities make them highly valuable for tissue engineering applications such as tissue regeneration, wound healing, drug delivery, and biosensors [117].

In this respect, an injectable hydrogel was created by a Schiff base reaction using catechol-modified chitosan and an aldehyde-modified nanocrystalline cellulose matrix. Double crosslinking was accomplished by inserting silver particles into the matrix via Schiff base bonds. The produced hydrogels demonstrated excellent mechanical performance, antibacterial characteristics, biocompatibility, and the ability to release silver particles in a controlled manner. Furthermore, the hydrogels remained firmly attached to irregular-shaped wounds, in vitro and in vivo testing revealed the ability to induce neovascularization and tissue regeneration [118]. Another investigation used the Schiff base reaction to crosslink dialdehyde carboxymethyl cellulose (DCMC) with gelatin. The resulting three-dimensional hydrogel exhibited a crosslinking degree of approximately 50, with a maximal swelling capacity of around 74 g/g at pH 10.0 and 37 °C, and it reached swelling equilibrium within three hours. Also, the hydrogel showed good mechanical properties, degrading 82.67% in a twelve-week period [119]. In this same context, Sheng et al. [120] prepared oxidized carboxymethyl cellulose crosslinked with polyacryloyl hydrazide via a Schiff base reaction in a physiological environment to produce injectable hydrogels. The gels exhibited a swelling ratio variation from 19 after seven hours to 28 after 20 days in a phosphate buffer (pH 7.4) at 37 °C, degrading only 47%. Furthermore, the hydrogel released 88% of the drug in vitro after 8 days.

(b) Crosslinking via Michael Addition: The Michael addition reaction forms a C–C bond between a carbanion or other nucleophile (such as amines or thiols) and an α,β-unsaturated carbonyl compound. This reaction is fast at room temperature, has low curing times, involves fewer toxic precursors, and does not require UV radiation, free radicals, or other crosslinking agents [121]. Furthermore, it is characterized by its high regioselectivity, efficiency, low reversibility, and absence of byproducts. There are two main variations: the aza–Michael addition (carbon–nitrogen bond) and the thio–Michael addition (carbon–sulfur bond), both applied in the synthesis of hydrogels, achieving a homogeneous and biocompatible polymer network.

It is possible to synthesize polyelectrolyte hydrogels by Michael addition, using divinylsulfone (DVS) as a crosslinker for the hydroxyl groups of cellulose and the carbon–carbon double bonds [122]. Since the thio–Michael addition is tolerant to air and water, it is ideal for the modification of polysaccharides, allowing mild, adaptable, and efficient reaction conditions with fast kinetics in water, making it a suitable method for obtaining polysaccharide hydrogels [123]. For instance, hydroxypropylcellulose and dextran were modified by methacrylation, and the resulting polysaccharide containing α,β-unsaturated esters was then chemically treated by the addition of thio–Michael with thiols, including cysteine, 2-thioethylamine, and quaternary ammonium salt functionalized with thiol groups. FT-IR studies showed the disappearance of the distinctive C=C stretching peaks at 1640 cm-1 after the thio–Michael reaction. This process presented advantages such as high and rapid conversion and water as a solvent. Hydrogels with quaternary ammonium salts demonstrated competitive antibacterial activity, suitable for biomimicry, antibacterial activity, and gene delivery applications [123].

Another example of this application is the preparation of a robust hydrogel by dissolving native cellulose in a cold alkaline solution (LiOH/urea) and then chemically crosslinking it with methylene bisacrylamide (MBA) via the Michael addition mechanism. This allowed hydrogels with different cellulose concentrations (2, 3, and 4 wt%) to be obtained and molar proportions of MBA/glucose to be tested (0.26, 0.53, and 1.05). The hydrogel was cured at 60 °C for 30 min, resulting in a water swelling capacity of 220 g H_2_O/g dry hydrogel, proving to be a rapid and straightforward alternative to prepare high molecular weight chemically crosslinked cellulose hydrogels [124].

### 3.3. Free Radical Polymerization

**Figure 7:**
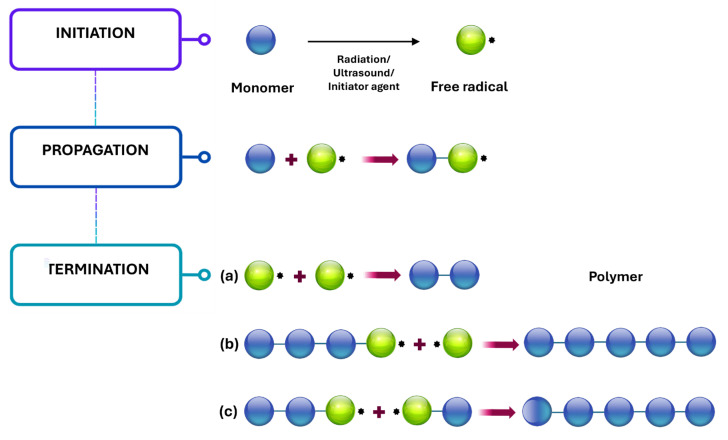
Radical polymerization steps. A physical or chemical initiator agent induces the formation of free radicals. These radicals can bond with monomers, leading to the formation of chains of varying lengths. The termination step occurs when two free radicals link together through various mechanisms: (a) the merger of two free radicals, (b) the interaction of a radical with another free radical within a chain, and (c) the joining of two distinct chains, each containing a free radical.

This process produces polymer chains by adding free radicals, as explained in Figure 7, a process known as chain-growth polymerization, which occurs in three main steps: initiation, propagation, and termination. Free radicals form during the initiation step through several mechanisms, including chemical initiators, various forms of radiation, thermal generation, and ultrasound. In the propagation stage, these radicals react with monomers, converting them into active forms that react with other monomers. The termination step may occur through either radical formation of polymeric matrices or chain transfer. Free radical addition copolymerization occurs when two or more unsaturated monomers polymerize. Even in moderate settings, this is a highly effective, popular, and rapid approach for hydrogel synthesis and can be used in bulk or as a solution. The choice of a crosslinker is primarily determined by the monomers and solvents that will be employed. Nonetheless, the number of crosslinkers used defines a specific hydrogel’s properties and swelling characteristics [125]. On this basis, stimuli-responsive hybrid hydrogels were prepared with succinylated cellulose nanocrystals and poly(N-isopropyl acrylamide) (PNIPAm) through free radical polymerization. The resulting hydrogels displayed pH and thermo-responsive behavior and were able to release famotidine when exposed to diverse pH values; they also showed a high response to temperature changes, decreasing their hydrophilicity and swelling ratio above 35° [126].

### 3.4. Thermal Induced Crosslinking

Initiation by thermal generation of free radicals from a chemical compound such as potassium persulfate occurs when the temperature reaches 70 °C, generating sulfate radicals, which further initiate reactions in the system [127]. For instance, Bao et al. [128] synthesized hydrogels by grafting acrylic acid/acrylamide/2-acrylamido-2-methyl-1-propanesulfonic acid onto sodium carboxymethyl cellulose (CMC) and montmorillonite by initiating the reaction with potassium persulfate and N, N′-methylenebisacrylamide as a crosslinking agent. A hydrogel with a porous structure and side chains was obtained, and its swelling properties depended on external pH, particle size, and salt concentration.

### 3.5. Photo-Induced Crosslinking

Polymer crosslinking can be induced by light irradiation of photosensitive materials present in a polymer solution, which typically respond to specific wavelengths of light. Sources usually include ultraviolet (UV) light or visible light. For instance, using ruthenium (Ru) compounds in the presence of blue light promotes the formation of covalent bonds between polymers [129]. In another instance, Silva et al. [130] developed innovative single-component, cellulose-based hydrogels for tissue engineering applications using Digital Light Processing (DLP) for 3D printing. This technique employs a digital light source to project light onto a photo-responsive solution, curing it layer by layer to create a 3D object. The hydrogels showed dimensional stability and could create high-resolution, free-standing 3D objects with precise shapes. Additionally, they demonstrated rapid curing kinetics, yielding hydrogels with robust mechanical properties capable of withstanding compressive stress. Biological assessments revealed that the hydrogels were resistant to hydrolytic degradation, retained their shape for up to four weeks, and displayed cytocompatibility with fibroblast cells, highlighting their potential for biomedical applications. Also, using this photo technique, Chen et al. [131] prepared injectable hydrogels for tissue engineering, reporting an improvement in the Young’s modulus in hydrogels synthesized by esterification of methacrylate groups linked to oxidized hydroxypropyl cellulose (Ox-HPC), followed by UV treatments. The findings showed minimal modification of the microporous structure of the hydrogel due to crosslinking, resulting in a stronger and less hydrophilic hydrogel. Furthermore, the hydrogel displayed injectability and self-healing qualities before and after UV treatment. In another study, a hydrogel for brain tissue engineering was achieved by brief in situ exposure to visible light, resulting in a photo-crosslinked hyaluronic acid methacrylate (HAMA) and carboxymethylcellulose methacrylate (CMCMA) hydrogel with various compositions (25%, 50%, and 75% *w*/*v*). The photo-initiator used was lithium phenyl-2,4,6-trimethylbenzoylphosphinate, and a 400 nm light source was employed. The produced hydrogel was biocompatible, mechanically robust, biodegradable, and suitable as an anti-adhesive agent and dural substitute. Subsequent in vivo studies showed a significant reduction in fibroblast penetration and adhesion and the potential to heal dural abnormalities while protecting the dura mater from postoperative adhesion [132].

### 3.6. Radiation-Induced Crosslinking

Radiation-induced crosslinking of polymer chains is an environmentally friendly process that allows the quick and efficient formation of homogeneous hydrogels in a single step. This process can be controlled by adjusting the intensity and dosage rate of the radiation, eliminating the need for catalysts or other chemical agents. High-energy radiation, such as gamma and electron beam irradiation, causes the formation of free radicals, peroxides, and hydroperoxides, which in turn promote or initiate polymerization and crosslinking reactions without requiring activation agents [133,134,135].

In this context, Hiroki et al. [136] prepared hydrogels by electron beam irradiation on aqueous solutions of hydroxypropyl cellulose, dimethacrylate, polyethylene glycol #1000, and 2-hydroxyethyl methacrylate. These hydrogels exhibited a tensile strength representing an approximately two-fold increase compared to a hydroxypropyl cellulose hydrogel. Likewise, the hydrogels were highly biodegradable and compatible with human eye tissue, making them suitable for soft contact lens fabrication due to their low protein adsorption.

On the other hand, cellulose-based hydrogels were prepared by radical polymerization using a solution of dimethylaminoethyl methacrylate and cellulose. The results showed a correlation between the degree of grafting and the crosslinking of the hydrogel, which increased with ionizing radiation dose (10, 30, or 100 kGy). The resulting hydrogels had a Young’s modulus between 14 and 39 kPa, which makes them suitable for biomedical applications. In addition, they incorporated silver particles into their structure and adsorbed Fe^3+^ ions [137].

### 3.7. Crosslinking via Condensation Polymerization

Condensation polymerization is a type of step-growth polymerization in which polymers form through condensation reactions that occur between at least two functional groups present in the monomers to be polymerized. A reactive group links monomers, creating a linear chain and releasing a byproduct. The process follows three steps: initiation, in which reactive species (initiator agent) react with a functional group in the monomer; creating an active center propagation where the active center reacts with another monomer extending the length of the chain, and termination is the last step in which there are no more actives centers thus stopping chain growth.

Sol–gel inorganic polymerization is a popular approach for synthesizing hybrid hydrogels by hydrolysis–condensation. It is a green technique since it does not need organic chemicals or solvents. The covalent network is formed by condensing a single function in the presence of a metal or metalloid-containing precursor (metal oxide or metal halide), with silicon alkoxides being the most used precursors. The reaction can be performed at ambient temperature and neutral pH in water, and its byproducts are usually water or low molecular alcohols (methanol, ethanol, and isopropanol) [138,139]. Furthermore, acidic or basic catalysts can be added to accelerate the reaction [140]. For example, a hydrogel was produced by combining sodium carboxymethylcellulose (CMC) and hydroxyethyl cellulose (HEC). In this case, HEC was used to promote intermolecular crosslinking since CMC had a low crosslinking degree because of its high degree of substitution and electrostatic repulsions between chains. The preparation method was simple; first, HEC was dissolved entirely in distilled water, and then CMC was added while stirring. The hydrogel was obtained by adding citric acid in various concentrations (1.75, 2.75, 3.75, 10, and 20% *w*/*w*) to produce hydrogels with different crosslinking degrees. The hydrogels could hold much water, and the method produced no toxic byproducts [141].

### 3.8. Ultrasound-Induced Crosslinking

Ultrasound with frequencies below 500 kHz has been used to create physical and chemical interactions and can be utilized for polymerization. Physical effects of low-frequency ultrasound include heat generation, high shear rates, and triggering the formation of free radicals in the solution. In the absence of oxygen and traditional free-radical initiators, low-frequency ultrasound generates free radicals in water, which help prepare hydrogels [142].

A sol–gel transition of carboxylated cellulose nanocrystals was investigated to determine the effects of ultrasound treatment. During the process, it was seen that cellulose fiber underwent fragmentation, and the sonication treatment induced a sol–gel transition. In addition, the gel state and the gelation process did not depend on the concentration of cations. However, it was found that cations must be added before sonication to produce firm hydrogels, and the length of the ultrasonic treatment determines their rheological properties [143].

### 3.9. Biologic Crosslinking

Another method for forming hydrogels is through an enzyme-catalyzed process. This approach is often used to produce injectable hydrogels in situ. The most commonly used enzyme catalysts for crosslinking hydrogels are transglutaminase, tyrosinase, and horseradish peroxidase [144]. These enzymes inhibit side reactions and exhibit high substrate selectivity. Transglutaminase, a calcium-dependent enzyme, catalyzes covalent crosslinking reactions by forming an amide bond between carboxamide groups and primary amines on polymers or polypeptides. Hydrogel production mediated by TG has been effectively applied in several systems, such as polypeptide hydrogels. Moreover, careful peptide sequence design can reduce gelation periods to a few minutes. Hydrogels produced by this method are employed as surgical tissue adhesives due to their strong adhesive qualities [145]. Horseradish peroxidase and water have been utilized to crosslink hydrogels based on HA, dextran, cellulose, and alginate. In this context, gelatin and nanocrystalline cellulose hybrid hydrogels were prepared. The physicochemical properties of the composite hydrogels demonstrated that the addition of nanocrystalline cellulose and crosslinking with transglutaminase significantly increased the breaking strength and storage modulus of gelatin hydrogels by 30 times at 25 °C compared to pure gelatin hydrogels. Additionally, their biocompatibility was high when evaluated with HeLa cells using the MTT method [146].

### 3.10. Hybrid Hydrogels

To improve hydrogels or to grant them unique properties such as mechanical, diffusion rates, hydrophilicity, and biological activity, among others, hybrid hydrogels have been engineered by incorporating different types of materials into a system. Complex systems are formed by combining natural and synthetic polymers, functional moieties, and particles, allowing tunable functions [147]. Hydrogels containing nano-sized materials can be developed by immobilizing different types of nanoparticles in various hydrogel compositions by either covalent or noncovalent bonds. Methods to homogenously distribute nanoparticles into the hydrogel structure include (a) directly forming the hydrogel in a nanoparticulated suspension; (b) physically embedding nanoparticles into a premade hydrogel de-swollen matrix by adsorption; (c) nanoparticle synthesis within a premade gel; (d) crosslinking hydrogels with nanoparticles; and (e) crosslinking a mixture of nanoparticles, polymers, and gelation agents [86]. Moreover, incorporating nanoparticles into hydrogels provides a new strategy to control and sustain the delivery of various hydrophobic therapeutic molecules by encapsulating them in nanoparticles such as micelles and liposomes [148].

## 4. Cellulose-Based Stimuli-Responsive Hydrogels

Stimuli-responsive hydrogels, usually dubbed smart or intelligent hydrogels, are materials that can rapidly change their morphology or properties in response to external stimuli, allowing for precise and controlled customization [149]. These hydrogels can release substances in targeted areas or provide mechanical support triggered even by slight changes. In addition, they have great potential in tissue engineering due to their versatility and ability to mimic natural biological processes. The phenomenon that causes molecular transitions in the networks of stimuli-responsive hydrogels is categorized as physical (light, temperature, electric or magnetic fields, pressure, or sound), chemical (humidity, pH, or ionic strength), or biological (enzymes or cells) [150]. Hydrogels can also be classified as single, dual, or multi-responsive materials based on how well they react to one, two, or more types of stimuli [151].

Some polymers, in their natural state, exhibit responsiveness to external stimuli, and researchers have used them alone or in combination with other materials to produce responsive hydrogels [152]. Likewise, they can be engineered by incorporating specific chemical moieties that undergo conformational changes in response to external stimuli [153]. A common strategy to produce stimuli-responsive cellulose and cellulose-derivative hydrogels is copolymerization with stimuli-responsive components.

In this framework, hydrogel’s responses may manifest by dynamic changes such as motion (twist, roll, bend, move, or fold), controlled degradation (crosslinking degradation, self-immolation, degradation), electrical charge change, state transition (solution to gel/gel to solution) or stimuli induced morphological changes that might be isotropic, increasing or reducing size (expansion/contraction, swelling/deswelling) or anisotropic, resulting in shape alterations due to linking or unlinking, tangling or detangling, assembly or disassembly, binding or unbinding. The anisotropic swelling or deswelling depends on the polymer used, polymer density, crosslinking agents, gradients, and layers or zones. In addition, transitions may trigger an expected action responding to a signal or stimulus such as drug delivery [153,154].

### 4.1. Hydrogels Responsive to Chemical Stimuli

Stimuli-responsive hydrogels may respond to chemical stimuli such as humidity, pH, or ionic strength by changing size or morphology, by degrading themselves, or by phase transition as described in Table 3 [152,153,154].

### 4.2. Ionic Strength and pH Responsive Hydrogels

pH-responsive hydrogels are made of polyelectrolytes with weakly acidic or basic groups in their structure that react to changes in the environmental pH by either accepting or releasing protons and they can be partially ionized due to the electrostatic forces of nearby ionized groups [155].

These hydrogels are categorized as either ionic or neutral based on their ionic charge. Ionic hydrogels are classified as anionic (negative charge), cationic (positive charge), or ampholytic (positive and negative charges) depending on the kind and quantity of ionic pendant groups included in their structure. Ionic hydrogels are sensitive to changes in pH in the environment and can abruptly alter their volume.

A cationic hydrogel holds basic groups like amines, while an anionic hydrogel has acidic groups like carboxylic acid and sulfonic acid. pH changes prompt these groups to receive or release protons, resulting in ionization and electrostatic repulsion, which modulates swelling behavior. When the pH of an ionizable group is higher than its pKa, anionic hydrogels ionize. In contrast, cationic hydrogels ionize in environments with a pH lower than the pKb of the ionizable species. Nevertheless, pendant groups of anionic hydrogels ionize at neutral pH and show maximum swelling at this pH [149,155,156,157].

Cellulose or cellulose-derivative hydrogels can be prepared considering that physical properties such as configuration, chain conformation, volume, and solubility can be designed by varying electrolyte concentration and the distribution of the charges along a polymer backbone. Generally, the pH range at which phase transition occurs can be modified by selecting an ionizable pendant group at a pKa corresponding to the target pH range or by incorporating hydrophobic groups into the polymer backbone [155].

In this regard, as the ionic strength rises, the swelling ratio of a pH-responsive hydrogel will decrease, since counterions in water weaken the electrostatic repulsion between adjacent polymer backbones. While the ionic strength of the external solution rises, water molecules from the hydrogel move towards the external solution to balance osmotic pressures, thus increasing the osmotic pressure difference between the exterior and interior of the hydrogel. As a result, hydrogels made of cationic polymers will expand at low pH levels and contract at high pH levels. For example, Arola et al. [158] observed that cellulose nanofibril hydrogels hardened and eventually flocculated as salt concentrations increased (NaCl, Na_2_SO_4_, NaI, NaSCN, and sodium acetate). The conclusion was that hydrogels with low cellulose nanofibril densities were very susceptible to salts; therefore, ions induce hydrogels to floculate even at very low solute concentrations, such as one mM.

Anionic polymers are hydrophilic when the pH value of the surrounding solution is greater than their pKa. In comparison, cationic polymers exhibit good solubility in water when the pH value of the external environment is lower than their pKa [159]. Likewise, the response rate increases by increasing the number of ionic groups and the pore size and decreasing the crosslinking density and size [160].

In this context, cellulose derivatives such as carboxymethyl cellulose, cellulose acetate, and ethyl cellulose have been studied and used to develop pH-responsive hydrogels. Hydrogels fabricated using carboxymethyl cellulose are common because they contain carboxylic acid groups (COOH) bound to some of the hydroxyl groups of the cellulose backbone. This carboxylic acid acts as an anionic group and may interact electrostatically with cations by protonation-deprotonation. When the pH is acidic, polymer–chain interactions become stronger than the solvent-polymer interactions due to the protonation of carboxyl groups and the strong hydrogen bonding interaction between them. In contrast, the ionized carboxyl groups induce electrostatic repulsions at alkaline pH levels, resulting in swelling [161]. Various polyelectrolytes having cationic groups, such as polymers containing imine and amine groups, may interact well with CMC, as can several metal cations, including silver, aluminum, lead, iron, and copper [161,162].

For instance, Khan et al. [163] synthesized and characterized pH-responsive gelatin–carboxymethyl cellulose hydrogels crosslinked with glutaraldehyde by free radical polymerization. The maximum swelling of the hydrogel co-occurred with the highest release of the drug at pH 1.2. In addition, the porosity and the gel fraction increased proportionally with the increase in polymer load, and it was observed that drug release depended on the composition of the hydrogels as much as on the pH of the release solution.

In another instance, pH and salt-responsive hydrogels were synthesized by crosslinking carboxymethyl cellulose and quaternized cellulose with epichlorohydrin (ECH) in a NaOH solution. All hydrogels responded well from pH 1 to 11 and shrank at pH 12. Also, the hydrogels demonstrated swelling responsiveness in CaCl_2_, NaCl, and FeCl_3_ solutions. It was also observed that higher concentrations of carboxymethyl cellulose in the hydrogel led to greater swelling due to increased water adsorption. In addition, quaternized cellulose was shown to be responsible for regulating the charge [164].

Cellulosic pH-responsive hydrogels can also be synthesized by integrating functional groups and linkers, including pH-responsive cationic and anionic polymers and pH-cleavable linkers, as shown in Table 4 [165,166]. On this basis, cellulosic injectable hydrogels were prepared by copolymerization of oxidized carboxymethyl cellulose (CMC–CHO), carboxymethyl cellulose (CMC-NH_2_), and pH-responsive poly (ethylene oxide)-block-poly (2-(diisopropylamino) ethyl methacrylate) (PEO-b-PDPA). The injectable hydrogel was constructed via a Schiff base reaction by copolymerizing PEO-b-PDPA micelles with CMC-NH2 or CMC–CHO. This system proved to be tunable, pH-responsive, and able to release Nile Red dye and doxorubicin in a slow, controlled manner while exhibiting tunable degradation and a high storage modulus [167]. In another investigation, a composite hydrogel able to mechanically adjust to varying pH levels was prepared by bacterial cellulose nanofibers oxidized with dialdehyde and copolymerized with chitosan. The composed hydrogel showed strong mechanical characteristics in an acidic environment and poor mechanical qualities in a basic environment. This is ascribed to the protonation of the amino groups in the chitosan chains. Under acidic conditions, the composite experienced an increase in osmotic pressure, improving its mechanical strength. Furthermore, this material exhibited remarkable three-dimensional stability, maintaining its volume irrespective of acidic or basic environments [168].

### 4.3. Thermal Responsive Hydrogels

Thermoresponsive hydrogels’ dynamic response is prompted by shifts in environmental temperature [152]. The temperature at which hydrogels experience a reversible phase transition is known as the critical solution temperature (CST), and it depends basically on three kinds of molecular interactions: polymer–polymer, polymer–water, and water–water [152,169,170]. On this basis, transition can take place at a lower critical solution temperature (LCST) or an upper critical solution temperature (UCST) [171]. Transition at the Lower critical solution temperature (LCST) occurs when polymers are soluble below the CST in an aqueous solution due to hydrogen bonding interactions with water molecules. At the same time, above the CST, they become insoluble due to the disruption of hydrogen–water bonds and hydrophobic polymer-polymer interaction [172]. Polymers in aqueous solution below the critical solution temperature (CST) have a coil structure but turn into an insoluble spherical shape upon heating. LCST is subject to molecular weight and the concentration of the polymers. The LCST generally rises by binding hydrophilic species and decreases by interaction with hydrophobic ones. On the other hand, UCST polymers swell poorly or are insoluble in water at temperatures below the CST. Still, above it, they are soluble due to a hydrophobic–hydrophilic transition [173,174]. Furthermore, the gelation point can be thermodynamically controlled by altering the polymer composition. The LCST of a hydrogel can be changed by copolymerization using hydrophilic or hydrophobic monomers to raise or decrease it, respectively. UCST behavior may also be altered by increasing the number of polar groups on the polymer chains or modifying the polymer’s molecular weight.

In this framework, hydrogels with cellulose and cellulose derivatives as key components may be synthesized with polymers and agents with inherent temperature-responsive properties. Some representative synthetic polymers are listed in Table 5 [159,175]. For instance, since carboxymethyl cellulose can form hydrogen bonds and dynamic noncovalent interactions with nearby polymer networks and metal ions. Pang et al. [176] successfully fabricated thermoresponsive, conductive hydrogels with excellent mechanical properties and the ability to self-heal by free radical polymerization of carboxymethyl cellulose (CMC) and acrylic acid crosslinked with aluminum chloride hexahydrate. The hydrogel consisted of two networks: The first network was formed by hydrogen bonds between hydroxyl and carboxyl groups of carboxymethyl cellulose and polyacrylic acid (PAA) chains, while the second network was formed by dual bonding between Al^3+^ ions and carboxylic groups of CMC and PAA. These networks contributed to the hydrogel’s thermosensitivity, strong mechanical, and self-healing properties. The hydrogel thermoresponse rose from 8.3 to 87.9 in the 35 to 85 °C range, ascribed to the induced mobility of CMC and PAA chains and conductive ions at high temperature. Moreover, the hydrogel exhibited a stronger thermal response when subjected to stretching (0–400%) or twisting (0–720°).

Thermoresponsive hydrogels have been successfully synthesized using cellulose derivatives such as methyl cellulose, ethyl hydroxyethyl cellulose, hydroxypropyl cellulose, and hydroxymethyl cellulose [149].

Methylcellulose is a thermoresponsive cellulose derivative described by Parvari et al. [177] as an “abnormal” inverse-freezing fluid. It is well soluble in water at low temperature due to the disruption of intra and interchain hydrogen bonding. It presents an LCST behavior and undergoes intermolecular interactions to form a turbid gel upon heating at 60–80 °C while returning to its original solution state upon cooling [152,178]. Mechanical properties and LCST of methylcellulose hydrogels can be adjusted by altering the concentration of the solution, the heating rate, and the molecular weight of the polymer [178,179]. For instance, grafting methylcellulose with N-isopropylacrylamide (NiPAAm) proved that a low percentage of methylcellulose lowered the LCST of pNiPAAm, while a higher content of methylcellulose increased it. Also, adding methylcellulose to NiPAAM resulted in no syneresis and improved the mechanical strength of the subsequent hydrogel [152].

In the same context, water-soluble and pH-stable polymer hydroxypropyl methylcellulose produces thermoreversible hydrogels that change volume in response to temperature fluctuations. They exhibit hydrophilic properties and swell in water when the temperature is below the LCST. However, they become hydrophobic due to dehydration when the temperature exceeds LCST, resulting in a substantially decreased volume [149,180].

The phase transition temperature (Tt) of hydroxypropyl methylcellulose is around 60 °C; nevertheless, it has been reported that it is possible to lower this temperature and control the swelling behavior by addition of ions and other chemical agents such as glycerol which promotes the formation of a hydrophobic region and reduces Tt [181].

Hydroxyethyl cellulose is another cellulose derivative that exhibits a high degree of water solubility across a wide temperature range, even in high-temperature regions where other nonionic chemically modified cellulose ethers such as methyl cellulose (MC) and hydroxypropyl methyl cellulose (HPMC) exhibit cloud points. From this context, Arai and Shikata [182] studied the mechanism behind the high solubility of HeC by dielectric spectrum measurement of the temperature dependence on the hydration number per glucopyranose unit (nH). Aqueous solutions with a different molar substitution number were heated at a set temperature range (10 to 70 °C), showing no cloud points. When compared to methyl and hydroxypropylmethyl cellulose, it was observed that the hydroxyethyl cellulose molecules exhibited far milder dehydration behavior with rising temperatures, and the nH values were significantly greater than the minimal critical nH value (about 5) that is needed to dissolve cellulose ethers in water. The study concluded that the hydroxyethyl cellulose thermodynamic response was mainly affected by group substitution.

Furthermore, ethyl hydroxyethyl cellulose is a non-ionic, amphiphilic, and thermosensitive polymer. Its structure is defined by the presence of a small number of hydrophobic side groups connected to the primary cellulosic backbone; it undertakes a phase separation above LCST (near 34 °C), forming low-toxic, hydrophobic aggregates that separate from water [149,179,183]. Reversible hydrogels synthesis requires heating semi-diluted aqueous solutions of ethyl hydroxyethyl cellulose at high temperatures in the presence of an ionic surfactant. During this process, formed aggregates link to each other, creating a network, and the charge in the surfactant generates electrostatic repulsions and chain swelling due to water retention. In contrast, a lack of surfactant causes a macroscopic phase separation. As a result, the gelation process is controlled by the equilibrium between linking and swelling. The most used ionic surfactants in the synthesis of ethyl hydroxyethyl cellulose hydrogels are sodium dodecyl sulfate (SDS) and cetyl trimethylammonium bromide (CTAB) [183,184].

Furthermore, comprehensive research has been conducted into developing temperature-sensitive hydrogels that include charged nanocellulose fibers to simultaneously substitute ionic surfactants and enhance their mechanical characteristics. For example, to avoid poor mechanical properties and toxicity in physically crosslinked ethyl hydroxyethyl cellulose thermoresponsive hydrogels, Dashtimoghadam et al. [183] examined the use of negatively charged cellulose nanofibers as a potential substitute for ionic surfactants. These nanofibers were found to induce inner electrostatic repulsions, resulting in the swelling degree required to produce a stable and non-toxic hydrogel at high temperature. In addition, it was discovered that the phase transition temperature of hydrogels (37 °C) was minimally affected by the quantity of cellulose nanofibers added. However, as the concentration of cellulose nanofibers increased, the strength parameter tripled.

Salts can modify the sol–gel transition temperature of a hydrogel. This occurrence may be explained by considering the Hofmeister order, which is a classification of ions based on their ability to absorb water and hence influence the stability of a solution. These ions are categorized as either structure formers (kosmotropes) or structure breakers (chaotropes) [184,185]. Kosmotropic ions exhibit a high degree of hydration, leading to an augmentation in hydrophobicity known as the “salt-out” phenomenon. On the other hand, chaotropic ions are weakly hydrated and enhance the solubility of nonpolar substances, resulting in a “salt-in” effect. Certain salts can either decrease or increase the sol–gel transition temperatures. The terms “salt-assisted” and “salt-suppressed” are employed to describe these sol–gel transitions, corresponding to the salt-out and salt-in effects, respectively [178].

For example, the transition temperature of salt-free methylcellulose from liquid to gel was 62 °C, whereas from gel to liquid was 32 °C. When a 0.8 M solution of NaCl was added, the transition temperature shifted to 43 °C and 28 °C, respectively [186].

### 4.4. Hydrogels Responsive to Mechanical Stimuli

Certain hydrogels may transduce mechanical inputs into targeted chemical modifications. Hydrogels routinely undergo forces of compression and elongation that generate supramolecular disturbance, morphological changes in chain alignment, or excimer disruption or generation. Mechanical stimulation of the polymeric network triggers either non-scissile or scissile chemical responses. Covalent bond-breaking reactions exist in scissile transformation, including ring-opening and selective cyclo-conversions. Contrarily, the non-scissile systems depend on conformational changes, non-covalent bond disruptions, and the formation or destruction of aggregates [151].

A method for producing stress-responsive hydrogels from cellulose and cellulose derivatives involves incorporating mechanophores, which undergo mechanically triggered reactions. The specific response may be customized based on the type, content, arrangement of attachment sites, and distribution of mechanophores within the polymer chain. In addition, many mechanophores exhibit a mechanochromic behavior, inducing changes in various chemical structures that manifest as a color change [187,188].

A hydroxypropyl cellulose solution containing less than 45 wt% water at ambient conditions can self-assemble into a cholesteric liquid crystal, and this photonic, liquid crystalline mesophase exhibits an exceptional mechanochromic response. Considering this, Barty-King et al. [189] formed a stress-responsive, supramolecular photonic hydrogel by aqueous dissolution of hydroxypropyl cellulose and gelatin. The hydrogel was observed to retain the mechanochromism, the structural coloration, and the non-Newtonian behavior displayed by the solution. Analysis of stress applied showed a linear mechanochromic response, and the mechanochromic recovery time of the gel was 69% shorter than the liquid system.

Another type of highly sensitive hydrogel to mechanical forces is constituted by supramolecular assemblies, which consist of self-organized complexes held together by non-covalent bonds. In these assemblies, the breakage of weak non-covalent bonds such as hydrogen bonding, metal-ligand coordination, electrostatic interaction, π–π interaction, host–guest interactions, and dipole–dipole interaction, triggers a restructuration resulting in self-assembly or self-healing behaviors [190].

For instance, a high self-healing hydrogel was reported by Hussain et al. [191]. The hydrogel was based on hydroxyethyl cellulose enhanced with Fe^3+^ ions by supramolecular interactions. The designed hydrogels exhibited exceptional mechanical properties, including a tensile stress of 3.50 MPa, a tensile strain of 1245%, a compression stress of 32 MPa, and a self-healing efficiency of about 98%.

Strain signals can be converted into recordable electrical signals. Therefore, Chen et al. [165] engineered a cellulose strain-responsive hydrogel by grafting acrylonitrile and acrylamide copolymers onto cellulose to be used in a strain sensor. The resulting hydrogel showed high responsiveness and stability for monitoring human activities. Mechanical properties such as ultra-stretchability (1730%), high elasticity (90%), outstanding tensile strength (160 kPa), toughness (1074.7 kJ/m^3^), and fatigue resistance were attributed to dipole–dipole and multiple hydrogen-bonding interactions in the network.

### 4.5. Photo Responsive Hydrogels

Photoresponsive hydrogels undergo chemical and physical microstructural changes upon light exposure. Light is an ideal external stimulus to control hydrogel responses because it can regulate molecular characteristics such as conjugation, charge, optical chirality, amphiphilicity, polarity, and conformation. It can be applied remotely and dosed for precise response levels. Polymers can respond to various wavelengths by changing properties like form, wettability, solubility, optical quality, conductivity, and adhesion.

Cellulose photoresponsive hydrogels can be synthesized by integrating photo-responsive functional groups or chromophores in their polymer chain. Chromophores are molecules that absorb particular wavelengths of light and, in doing so, undergo reversible or irreversible reactions such as dimerization, isomerization, or cleavage, generating changes in the hydrogel conformation. The response depends on the type of chromophore, exposure time, light intensity, and wavelength [151,192].

**Figure 8:**
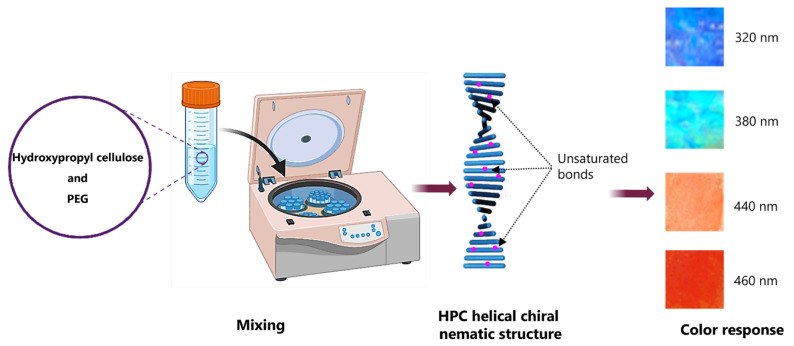
Mixing HPC and PEG to create a chiral nematic hydrogel with unsaturated bonds causes polar responses to stimuli by changing its structure and, therefore, showing color transitions depending on the distance between its plates.

For example, hydrogels with photo-switchable properties for drug release were prepared by formation of a host–guest complex between azobenzene-grafted carboxymethyl cellulose and β-cyclodextrin dimers crosslinked by disulfide bonds with agarose. The resulting hydrogels showed gel–sol phase transition response to reducing agents and ultraviolet light (UV), whereas self-healing properties were prompted by host–guest complexation. Its photoresponsive properties depended on the photo-isomerization of azobenzene, converting from trans (450 nm) to cis (365 nm) CD-dimers. Furthermore, it was observed that exposure to a reducing agent or UV radiation significantly increased the rate of drug release by up to 80% during a span of 3 h [193]. An exploration of an innovative approach to creating a unique hydrogel that was able to generate stable cholesteric liquid crystal structures exhibiting different structural colors was crafted using hydroxypropyl cellulose (HPC) and specific polyethylene glycol (PEG) guest molecules strategically positioned on the HPC helical backbone through electrostatic repulsion as shown in Figure 8 [194]. The polarity of PEG finely tuned the gel’s reflective color, while its high sensitivity to temperature, pressure, and stretching led to rapid, continuous, and reversible color changes. The remarkable properties of this chiral nematic gel make it well-suited for a diverse range of applications, including displays, wearables, flexible electronics, health monitoring, and multifunctional sensors [194].

Furthermore, photo-responsive hydrogels may be used to create intelligent medical devices. For example, a hydrogel composed of oxidized hydroxyethyl cellulose and allyl was developed for smart contact lenses. Oxidized hydroxyethyl cellulose molecular chains were used as biomacromolecule templates to form Schiff base borate and hydrogen bonds. A double bond functionalized spirooxazine (allyl spirooxazine derivative) was integrated to endow photo and pH sensitivity. The resulting hydrogel portrayed fast recovery with almost no hysteresis, outstanding compressive capacity, a percentage of visible light transmittance around 93%, good pH adaptability, and no cytotoxicity in an in vitro test [192]. Photoresponsiveness may also be achieved by integrating nanomaterials that absorb near-infrared light in thermoresponsive hydrogels [193]. For instance, a photothermal intra-articular injectable hydrogel was investigated. The hydrogel formulation included methylcellulose, polypyrrole–polyethylenimine nanoparticles as photothermal agents, sodium chloride, and strontium ranelate to improve mechanical resistance and minimize gelation time. Analysis of the hydrogel demonstrated its capacity for sustained release of strontium ranelate while exhibiting exceptional mechanical strength. Moreover, the hydrogel system was biocompatible and could maintain cell viability and inhibit inflammation [194].

### 4.6. Magneto-Electro-Responsive Hydrogels

Hydrogels that respond to magnetic or electric fields have various biomedical applications, such as sensors, delivery of hydrophobic drugs and plasmonic nanomaterials, tissue engineering, and soft robots. They must possess a fast response, reversibility, safety, biocompatibility, and mechanical strength. Presently, significant research is being conducted to address limitations related to their composition, physicochemical and mechanical characteristics, and materials synthesis aimed at inducing specific responses to magnetic or electric fields. This class of hydrogels can alter their geometric dimensions and morphologies by expanding or contracting in response to an applied magnetic or electric field. The mechanism for induced deformation involves a combination of electrophoretic, electroosmotic, and Coulombic interactions [195]. Additionally, the production of cellulose-based hybrid hydrogels sensitive to magnetic or electric stimuli often involves the inclusion of conductive polymers and other electroactive components [196]. Some polymers that deform upon electric stimulation include chitosan, polyacrylic (PAAc), 4-hydroxybutyl acrylate (4-HBA), and poly(2-acrylamido-2-methylpropane-sulfonic acid) (PAMPS) [195]. Additionally, conducting polymers, including polypyrrole (PPy), polyaniline (PANI), and poly-(3,4-ethylenedioxythiophene) (PEDOT), are often used to produce hydrogels with electrical properties [197]. For example, electroactive hydrogels made of carboxymethyl cellulose, polythiophene, and acid-hydrolyzed cellulose (CMC/PTh/AHC) were tested for electroresponsiveness using bending sensitivity and angle measurements. The hydrogels bent toward a cathode electrode when an electric field was introduced. It was also discovered that the electroactive performance of hydrogels decreased when the AHC concentration rose. Among the hydrogels examined, the one with 10 wt.% of AHC exhibited the quickest induction time. The hydrogel with the highest self-healing efficiency in terms of tensile strength and elongation at break contained two wt.% of AHC, and it returned to its original condition after 24 h of healing. In addition, results indicated that this hydrogel had potential as an electrically stimulated actuator or an artificial muscle [198].

In addition, cellulose hydrogels may acquire magnetic responsiveness by incorporating magnetic particles into their polymeric networks and crosslinking them. An alternative method involves the precipitation of magnetic particles into the polymeric network before, during, or after the crosslinking process [199]. Fe_2_O_3_, Fe_3_O_4_, and CoFe_2_O_4_ particles are commonly used to prepare magnetic-responsive polysaccharide hydrogels. These magnetic hydrogels possess robust mechanical durability and stability under physiological settings, making them suitable for application in precise, sensitive biodevices for targeted drug delivery, tissue engineering, and hypothermia therapy [200]. For instance, a superparamagnetic hydrogel was created by freeze–thawing a mixture of poly(vinyl alcohol) and tricarboxy cellulose (CO) loaded with stabilized iron oxide particles, without using a crosslinking agent. The magnetic hydrogel exhibited a very effective distribution of magnetic particles throughout the matrix, while results from the Vibrating Sample Magnetometer indicated superparamagnetic properties [201].

### 4.7. Glucose-Sensitive Hydrogels

Glucose-sensitive hydrogels are used to create glucose sensors. These hydrogels can measure glucose levels and regulate insulin release in proportion to the glucose concentration, enabling continuous monitoring of blood glucose levels. Glucose sensors commonly used are Glucose oxidase (GOx), phenylboronic acid and its derivatives (PBAs), and Concanavalin A (Con A) [202].

Glucose oxidase is the most commonly used glucose sensor. It is an enzyme that is stable within a physiological pH range, being most stable at a pH value of 5, and it begins to degrade below two and above 8. Glucose reacts with the oxygen present in GOx, forming gluconic acid and hydrogen peroxide, thereby reducing the pH of the surrounding environment [203].

When glucose oxidase is coupled with a cationic pH-responsive hydrogel loaded with insulin, the pH decrease causes the hydrogel to swell and collapse suddenly, releasing insulin [202]. Due to the ability of the system to swell and deswell, insulin can be administered in a controlled manner. Under typical glucose levels, the hydrogel may release a standard amount of insulin. In contrast, the gel may collapse under elevated glucose levels and release a larger quantity. In this respect, a self-healing glucose-responsive hydrogel consisting of oxidized carboxymethyl cellulose (OCMC) and carboxymethyl chitosan (CMCS) was loaded with gold nanoclusters and glucose oxidase as fluorescent probes. The hydrogel demonstrated exceptional biocompatibility, self-healing, and sensitivity to cover the clinical detection range of glucose. Fluorescence studies of the hydrogel in diabetic mice and in vitro blood fluorescence tests further demonstrated its potential use in implanted biosensing for glucose monitoring [204].

Phenylboronic acid (PBA) is a versatile chemical that forms reversible bonds with glucose. Its advantages include long-lasting glucose affinity, simultaneous glucose level detection and insulin release, non-toxicity, and high stability [205]. Furthermore, cellulose-based, biocompatible glucose-responsive hydrogels can be designed by introducing a phenylboronic acid group in a cellulose hydrogel matrix. For example, a cellulose/4-vinyl-phenylboronic acid hydrogel was prepared using electron beam irradiation. The dual-crosslinked network was loaded with insulin labeled with fluorescein isothiocyanate. Analysis of the swollen hydrogels found glucose content and response to a glucose threshold by insulin release [206].

Concanavalin A (ConA) is another compound commonly used for glucose sensing. ConA is a non-sugar lectin derived from the jack bean (Canavalia ensiformis) with a reversible affinity for pyranose rings of glycopolymers, monosaccharides, and polysaccharides such as D-glucose. Glucose-sensitive cellulose hydrogels are obtained by binding ConA to the glycosylated groups of a hydrogel, forming a structured matrix with small pore diameters that trap insulin. Due to its higher affinity for ConA compared to glycosylated groups, glucose may displace the glycosylated moieties, resulting in either swelling of the hydrogel or an inverse sol–gel transition, leading to the release of insulin [159,202]. For example, Lin et al. [207] used carboxylated pullulan and covalently modified concanavalin to create glucose-sensitive hydrogels by moderate chemical activation. Subsequently, the hydrogels were loaded with insulin by in situ absorption. A scanning electron microscopy (SEM) revealed that the hydrogels exhibited crosslinked network architectures with evenly distributed pores and embedded insulin particles. Similarly, hydrogel release performance proved their ability to fast deliver insulin in a regulated manner as a response to variations in glucose levels. This poses them as promising materials for artificial pancreas systems.

### 4.8. Enzyme-Responsive Hydrogels

Cellulosic enzyme-responsive hydrogels possess the proper macromolecular networks to undergo controlled transitions and release substances they hold and protect to specific locations, under regulated conditions, when exposed to particular enzymes. These enzymatically responsive systems have potential applications in drug delivery, tissue engineering, regenerative medicine, cell culture, biosensors, smart antibacterial treatment, advanced diagnostic systems, scaffolds, wound dressings, and smart bandages, among others. At present, research is ongoing to engineer cellulose-based hydrogels with triggered enzymatic self-assembly properties for specific applications that can overcome interference from other biological systems, allow self-assembly under constant conditions, spatiotemporal controlled growth, and mechanical efficiency among many others [208].

The most common approach for designing cellulose-based enzyme-responsive hydrogels involves enzymatic breakdown, where a specialized enzyme degrades a hydrogel network by identifying and breaking down peptide chains incorporated as linkers or crosslinkers [159]. In addition to acting as organic triggers, enzymes can facilitate the controlled and prolonged release of bioactive substances through enzymatic movement. Enzyme-sensitive hydrogels consist of two primary functional components: an enzyme that recognizes and has access to a substrate, and a functional element that regulates molecular interactions and induces macroscopic transitions in a hydrogel. The macroscopic transitions are significantly influenced by hydrogen bonding, van der Waals forces, electrostatic, π–π, and hydrophobic molecular interactions, and they manifest as changes in the surface structure, swelling, or deswelling [209]. On this basis, Baretta et al. [210] reported an enzyme–nanozyme cellulose-based hydrogel prepared via a one-pot approach for the application in cascade catalysis. The integrated system simultaneously immobilized Prussian blue nanoparticles and active glucose oxidase in a sodium carboxymethyl cellulose matrix. The hydrogel matrix provided a biocompatible network for enzyme immobilization without affecting catalytic activity. Prussian blue nanoparticles functioned as both a network crosslinker and a peroxidase-like nanozyme. When assessed, the hydrogel displayed enhanced colorimetric glucose detection and improved stability compared to carboxymethyl cellulose hydrogels containing glucose oxidase or Prussian blue. Additionally, the ability of the colorimetric assay was validated for glucose detection in human serum samples.

### 4.9. Multi-Responsive Hydrogels

Hydrogels can be expertly engineered to respond to multiple stimuli simultaneously. This remarkable adaptability enables them to perform various functions, catering to the precise demands of various tissue engineering applications in response to specific physiological triggers and changing their properties when exposed to particular environmental alterations. This dual responsiveness enhances their effectiveness and opens up new possibilities in tailored medical treatments and responsive materials, ensuring they meet the dynamic challenges presented by their surroundings [211]. For instance, Zhou et al. [212] designed injectable hydrogels with both pH and self-healing responsiveness for tissue engineering applications. The hydrogels were prepared by reacting oxidized hydroxypropyl cellulose (Ox-HPC) with carboxymethyl chitosan (CMCS) via a Schiff base reaction, creating reversible imine bond crosslinks. The obtained hydrogels showed injectable and self-healing responsive properties. When Phenylalanine was used as a model amine-containing drug, it was released faster at a pH of 6.8 than at a pH of 7.4. In a separate study, a thermo-pH-responsive injectable hydrogel was developed for bone regeneration [213]. This hydrogel was created using chitosan reinforced with a blend of cellulose nanofibers and nanocrystals. The chitosan solution underwent a sol–gel transition at body temperature, while including the nanocellulose mixture enhanced its mechanical properties and significantly improved the gelation kinetics. Analysis through live-dead cell staining demonstrated high cell viability, indicating the biocompatibility of the hydrogels. Furthermore, cellulose nanocrystals (CNCs) induced a spreading cell morphology due to increased mechanical sensitivity. Multifunctional, multiresponsive hydrogels are presently being designed for skin regeneration. For instance, supramolecular hydrogels with injectable, self-healing, and antibacterial responses were developed by combining phenylazo-terminated Pluronic F127, quaternized chitosan–graft–cyclodextrin, and polydopamine-coated tunicate cellulose nanocrystals [214]. This blend was processed under physiological conditions through photoisomerizing azobenzene at different wavelengths, resulting in a supramolecular hydrogel characterized by a variable crosslink density within its network. Schiff base bonds and hydrogen bonds effectively hindered the complete gel–sol transition. The hydrogels demonstrated inherent antibacterial properties, favorable drug release behaviors, self-healing capabilities, hemostatic performance, and biocompatibility. Additionally, curcumin-loaded hydrogels exhibited multi-responsive release profiles in response to light, pH, and temperature variations. 

## 5. Cellulose-Based Hydrogels for Tissue Engineering

**Figure 9:**
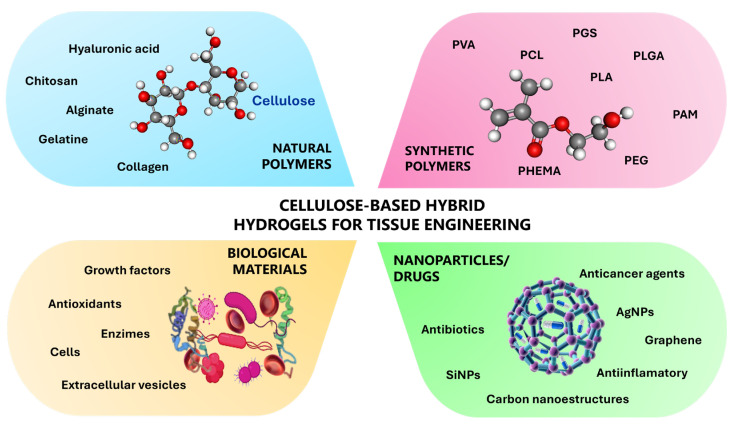
Cellulose-based hydrogels can be functionalized by copolymerization with other bio or synthetic polymers, the addition of growth factor or other biological material, and nanoparticles to fulfill tissue engineering requirements.

Tissue engineering is a multidisciplinary field combining engineering and science principles to create functional biological tissues or organs. It involves the development of biomaterials in combination with growth factors and cells to repair, regenerate, or replace damaged tissues and organs in the human body [21]. Various challenges exist when creating materials for tissue engineering, such as biocompatibility, cell attachment and proliferation, nutrient diffusion, degradability, clinical translation, and large-scale production. Biomaterials offer significant benefits, including high biocompatibility, cost-effectiveness, sustainability, and minimal toxic or side effects. Hybrid hydrogels can fulfill these needs by adding materials and agents that may create or enhance desirable characteristics (see Figure 9). However, some biomaterials still encounter challenges related to degradation, porosity, wettability, mechanical strength, and potential immunogenicity [215]. In this context, hydrogels derived from cellulose and its derivatives exhibit unique characteristics, including versatility, functionalization, adjustable mechanical properties, hydrophilicity, biocompatibility, biodegradability, porosity and high specific surface area; thus, finding use across several domains associated with tissue engineering due to their ability to emulate the inherent characteristics of the original extracellular matrix (ECM) a structure that dynamically holds together cells and organs along with the regulation of critical cellular processes such as differentiation, adhesion, migration, and polarization [216]. These hydrogels may be customized to closely replicate the structural and mechanical properties and even the response of native tissues, facilitating efficient mass transfer, promoting cell adhesion, and supporting protein functionality. Since cellulose-derived hydrogels can be designed to possess injectability and flexible structure, they can adjust well to the uneven geometry of injuries and wounds. Furthermore, their ability to facilitate liquid transference renders them appropriate substrates for transporting drugs and cells to specific locations with little intrusiveness. Cellulose-derived hydrogels have attracted increasing attention as they provide safer and more efficient alternatives and solutions to issues related to synthetic materials, such as infection risk, implant extrusion, and foreign body response [217,218,219].

They also offer several advantages for tissue engineering, including high hydrophilicity, biocompatibility, a 3D network structure, high degree of swelling, ample space for cells to grow and proliferate, adjustable mechanical properties to ensure stability and support for tissue growth, and they are suitable for cell encapsulation and controlled release [201].

### 5.1. Culture of Pluripotent Stem Cells

Remarkably, human embryonic stem cells (hESCs) and human induced pluripotent stem cells (hiPSCs) can self-renew and differentiate into the different cell types that form the human body. These cells, therefore, show outstanding potential for applications in tissue engineering. Nevertheless, at the present time, the in vitro two-dimensional culture techniques are incapable of accurately replicating the three-dimensional in vivo environment of the cell niche. This niche temporarily supports stem cell proliferation and undergoes dynamic modifications that promote future differentiation throughout the developmental process. However, failure to perform this vital function may result in polarization and abnormal cell morphology, which can reduce stability, shorten the lifespan of cells, and even result in cell death. In this respect, cellulose hydrogels are three-dimensional structures that provide a mimetic cell niche environment, allowing cell proliferation due to their biocompatibility and ability to fine-tune their morphology and mechanical properties. In this regard, it is possible to cultivate pluripotent stem cells by either microencapsulation or unrestricted proliferation within a large hydrogel. Hydrogels also support the mass transport of soluble molecules, nutrients, and metabolic byproducts in vivo or in vitro by exhibiting the appropriate hardness, hydrophilicity, porosity, and thickness [220]. From this perspective, Hao et al. [220] cultured induced pluripotent stem cells (hiPSCs) in nano-fibrillar cellulose hydrogels with different thicknesses to investigate the effect of the hydrogel property on temporal-spatial heterogeneity. Hydrogels demonstrated enhanced mass transfer capabilities due to their structure’s interconnected macropores and micropores. After five days, over 85% of cells at different depths remained viable in a 3.5 mm-thick hydrogel. Loss of pluripotency was observed at the bottom of the hydrogel, and the buildup of lactic acid over time caused pH changes, altering the charge of cellulose and its growth factor potential.

### 5.2. Cartilage Tissue Engineering

Cartilage is a connective tissue consisting of collagen type II and aggrecan (e.g., ribs and synovial joints), collagen type I (e.g., intervertebral disks), collagen type II, elastic fibers, and proteoglycans (e.g., ear and larynx) produced by cells known as chondrocytes. After being synthesized, cartilage is devoid of blood or lymphatic supply, and it is surrounded by a perichondrium membrane, making it difficult or impossible to mend itself after injury or illness [221].

In this context, hydrogels can be designed to exhibit mechanical, swelling, and lubricating properties comparable to articular cartilage. Their viscoelastic properties enable the distribution of mechanical stress and promote the growth of loaded chondrogenic cells into their characteristic spherical shape. Moreover, they can be tailored to respond dynamically to external stimuli, allowing for the control and monitoring of body functions. In this regard, aiming to replicate cartilage’s natural structure and function, extensive research has been conducted on cell-laden hydrogel bioinks for 3D cartilage bioprinting [222]. However, one of the ongoing challenges with three-dimensional hydrogel scaffolds is their need for substantial mechanical qualities. Therefore, Boyer et al. [223] developed a composite hydrogel nano-reinforced with laponites to increase its mechanical properties. In vitro investigations showed that laponites were cytocompatible and allowed oxygen diffusion within the network. Next, a hybrid scaffold prepared with laponite hydrogel and chondrogenic cells was studied in vivo by implanting it in subcutaneous pockets of nude mice for six weeks. Histological analysis of the scaffold revealed cartilage-like tissue formation and an extracellular matrix made of collagens and glycosaminoglycans, thus proving the cellulose-based hydrogel’s potential for treating cartilage defects. 

Another essential characteristic of cellulose hydrogels for cartilage is their hydrated nature, similar to the extracellular matrix (ECM). However, high content in water decreases hydrogels’ mechanical properties. To address this matter, hydrogels have been reinforced with fibers or particles to reduce hydrophilicity and increase mechanical strength. For instance, microcrystalline cellulose (MCC) hydrogels were synthesized by a multistep chemical and physical crosslinking to create a double network. The resulting macroporous structure and substantial water content created an ideal chondrocyte adhesion and growth matrix. Mechanical evaluation exhibited high compressive strength, higher compression recovery, and fatigue resistance compared to single-network hydrogels. It was also found that safe and non-toxic, double-network cellulose hydrogels displayed a strong histocompatibility by in vivo implantation, long-term stability, and slow degradation [224]. In another instance, cellulose nanocrystals reinforced collagen-based hydrogels to control hydration and improve mechanical properties. The composed hydrogels underwent plastic compression to remove excess liquid. It showed improved mechanical characteristics and increased mesenchymal stem cells’ chondrogenic differentiation when tested. The plastic compression of the hydrogel positively impacted the viability of encapsulated mesenchymal stem cells after seven days, developing good morphology and secretion of glycosaminoglycans [225].

Buchtová et al. [226] investigated water behavior inside injectable silanized hydroxypropyl–methylcellulose (Si-HPMC) hydrogels reinforced with 2 and 3 wt% of mesoporous silica nanofibers. The structure of the polymer reinforced with 2 wt% of Si presented a highly porous morphology, whereas three wt% of Si enhanced the mechanical properties of the matrix. Overall, it was shown that high water dynamics in this material supported nutrition, transfer, and cell nurturing, making them suitable for application to intervertebral disks, cartilage, or fibrillated heart tissue, and it could be considered for micro-invasive surgery.

### 5.3. Skin Tissue Engineering

Skin is the largest organ of the body; it is a barrier that plays several essential functions, such as protection against chemical, physical, and biological factors, metabolic and immunological processes, thermoregulation, and adjustment of the homeostatic equilibrium [227]. When injured or damaged, a complex healing process involves the activity of chemokines, cytokines, growth factors, regulatory molecules, resident skin cells, the extracellular matrix, and peripheral blood mononuclear cells [228]. However, severe injuries or systemic disease can damage the skin to the point that it cannot recover by itself or could ultimately be lost. Therefore, skin tissue engineering has focused on developing skin substitutes to regenerate or replace injured or damaged skin. Among those skin substitutes, cellulose-based hydrogels are very promising materials that resemble the structural and functional characteristics of the skin due to their biocompatible nature and ability to hold cells and substances involved in the skin healing process. For instance, Bektas et al. [229] designed a bilayer skin graft for the treatment of chronic wounds formed by a first layer of sodium carboxymethyl cellulose loaded with fibroblasts and a second layer made with either collagen or chondroitin sulfate-incorporated collagen loaded with keratinocytes. The bilayer structure allowed transference between the two layers and paracrine signaling. It also showed porous interconnection, high water absorption, proper stability, and good elastic moduli. In addition, it supported the production of collagen I, collagen III, transglutaminase, and laminin, and the expression of basic-fibroblast growth factor (bFGF), vascular endothelial growth factor (VEGF), and Interleukin 8 (IL-8), stimulating the attachment and proliferation of cells on both layers. Fibroblast attachment and proliferation on the carboxymethyl cellulose layer were lower than keratinocytes on the collagen layer, in which a keratinocyte-dense and stratified epidermis-like layer was formed. The hydrogel was able to mimic skin tissue in vitro. Koivunotko et al. [230] prepared nanofibrillated cellulose (NFC) hydrogels doped with cellulase to carry platelet-rich plasma, a growth factor resource for skin tissue engineering. The hydrogel was studied in vitro and in vivo in a full-thickness excisional wound model in an SKH1 mouse. It was discovered that hydrogels containing PRP or cellulase did not impair normal cell function in vitro, and cellulase efficiently disintegrated cellulose. In vitro, NFC hydrogels slowed the rate of fibroblast migration. In vivo, the doped NFC hydrogel enhanced re-epithelialization and promoted collagen deposition. Additionally, after cellulase degradation of the NFC, PRP release stimulated angiogenesis, causing normal host reactions and applications. Palem et al. [231] fabricated a hydrogel based on carboxymethyl cellulose–agarose–polyvinylpyrrolidone loaded with in situ formed zinc oxide nanostructures (ZnO NS) by a one-pot method. In vitro biocompatibility assays with skin fibroblast (CCD-986sk) cells showed that ZnO NS facilitated cell adhesion and proliferation. Furthermore, the hydrogel increased cellular interactions and showed physicochemical, antibacterial, and biological properties. Additionally, an in vivo investigation demonstrated that the hydrogel system promoted faster wound healing after 18 days, outperforming unmodified CAP hydrogels.

Bacterial cellulose is the most widely used type of nanocellulose for reconstructing skin. It is a hydrogel containing nanofibrils that mimic the fibrillar component of the natural extracellular matrix. It also has a great capacity to retain moisture and exhibits appropriate mechanical properties, such as strength, Young’s modulus, elasticity, and conformability [232]. Utoui et al. [233] prepared bacterial cellulose crosslinked with polydopamine (PDA) hydrogels. The obtained hydrogels showed excellent tensile strength and wettability. As a matrix for cultivating murine NIH/3T3 fibroblasts, the hydrogels increased cytocompatibility, leading to better metabolic activity and proliferation and improved cell viability. Therefore, they can be substitutes for skin tissue and wound-dressing applications. Hosseini et al. [234] prepared carboxymethyl cellulose/sodium alginate-simvastatin hydrogels to enhance and accelerate skin regeneration of chronic wounds. These hydrogels prepared with different simvastatin concentrations showed a suitable swelling ratio, sound water vapor transmission, and degradation rates. Simvastatin was released in 5 days from the hydrogel matrix, improving the proliferation of skin cells. The in vitro results proved that hydrogels promoted controlled pro-inflammatory responses and enhanced secretion of anti-inflammatory cytokines compared to hydrogels with no SIM. Likewise, the hydrogels facilitated rapid healing in a full-thickness wound in a rat model, showing approximately 93% wound healing ratio against 58% performed by the control after 14 days post-surgery. Budharaju et al. [235] developed hybrid scaffolds to promote full-thickness wound healing with re-epithelialization and epidermis closure. The scaffolds were poly(3-hydroxybutyrate-co-3-hydroxyvalerate) nanofibers embedded with carboxymethyl cellulose/agarose (2:1) hydrogel. It was noted that incorporating the hydrogel into the nanofibers enhanced their mechanical strength. Cytocompatibility studies showed more than 75% of cell viability within the scaffolds. Additionally, a significant increase in adhesion, cell proliferation, and viability was observed in studies involving human adult immortalized keratinocytes (HaCaT), human adult dermal fibroblasts (HDFa), and rat full-thickness wounds.

**Figure 10:**
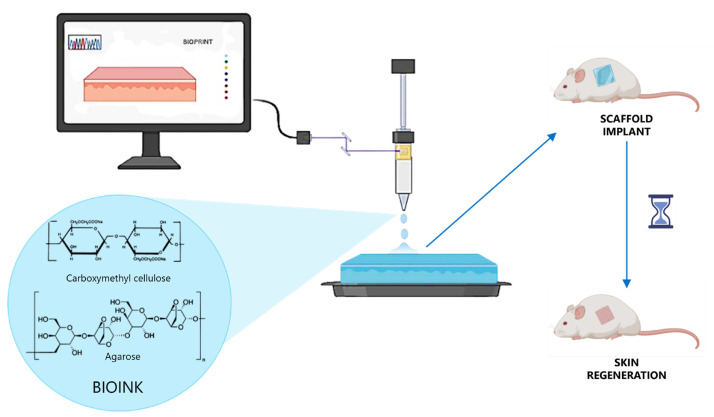
Cellulose-based hydrogels used for bioprinting human body tissue and evaluation of the printed scaffold by implantation in rats.

Tissue-engineered skin grafts have been the most effective treatment for significant skin defects. With the advent of 3D bioprinting, the microenvironment, the required shape, and structure of artificial skin replicas can be manufactured by computer-assisted deposition, as shown in Figure 10. However, the matrix material used as bioink for 3D printing artificial skin usually presents several challenges, such as proper mechanical properties and biocompatibility. In this context, Damle et al. [236] produced a biologically functional bioink for skin 3D printing based on an extracellular matrix comprising digested chicken skin, polyvinyl alcohol, and gelatin collagen. Cellulose-based hydrogels are extensively used as bioinks for 3D bioprinting for tissue engineering. In this sense, Budharaju et al. [237] prepared a thermoresponsive carboxymethyl cellulose/agarose bioink in 9:1, 8:2, 7:3, 6:4, and 5:5 ratios. The 5:5 ratio mixture formed a better gel at 37 °C, and its cytocompatibility was proved by in vitro extract utilizing skin fibroblasts. The 3D structures of 5:5 bioink structures were successfully extruded, and the printed structure maintained over 80% cell viability for seven days. In vivo studies in rat full-thickness wounds showed that both bulk and printed gels promoted skin regeneration.

### 5.4. Bone Tissue Engineering

Bone tissue engineering is a field that aims to develop strategies to regenerate, repair, or replace damaged or missing bone tissue using a combination of scaffolds, cells, and growth factors. From this perspective, cellulose and its derivatives present multiple advantages due to their non-toxicity, biocompatibility, biodegradability, availability, and low cost of production and processing, for bone damage treatment and they have been widely employed to transport growth factors and antibiotics directly to the location of damaged bone tissue to stimulate tissue healing [21]. The most popular cellulose derivatives for bone hydrogel scaffolds are carboxymethyl, methyl, and bacterial cellulose [238,239,240], and typically, cellulose-based hydrogels for bone tissue are mineralized, forming apatite and hydroxyapatite [238,239,240]. For instance, Niknafs et al. [241] developed hydrogels that included bioactive glass nanoparticles loaded into a matrix of bacterial cellulose and silk fibroin. They found that incorporating bacterial cellulose and nanoparticles into silk fibroin decreased the swelling rate and enhanced their mechanical properties while lowering their pore size. Also, according to gene expression experiments, hydrogel scaffolds improved cell adhesion, survivability, and differentiation [240]. Hybrid hydrogels that combine metal ions or are fabricated with metal particles have been the focus of recent studies, and they show great potential due to outstanding physicochemical properties and significant osteoinductive and osteogenic abilities [242]. In this context, a scaffold made from cellulose and hybrid mineralization of calcium and zinc was created to mimic the network structure of the extracellular matrix. This was achieved through the selective oxidation of bacterial cellulose, pre-mineralization, and in situ mineralization with calcium, zinc, and phosphate ions. In vitro experiments showed that the scaffold had no cytotoxic effects and significantly improved the adhesion and growth of bone marrow mesenchymal stromal cells. It also promoted osteogenic differentiation and demonstrated immunomodulatory properties by modulating macrophages. In vivo evaluation confirmed superior bone repair effects compared to other scaffolds [243].

The unique properties of bacterial cellulose, such as its high porosity, biocompatibility, good water retention, exceptional mechanical strength, and large surface area, make it an excellent hydrogel for bone tissue engineering. However, it does not have inherent osteogenic activity and cannot undergo biomineralization. In this respect, a bone-like apatite was formed in a bacterial cellulose-acrylamide hydrogel crosslinked by free radical polymerization with bis [2-methacryloyloxy] ethyl phosphate (BMEP). The hydrogels showed excellent compressive mechanical properties, highly interconnected porous structures, good swelling, biodegradability, increased BMEP content, improved fibrous structure, porosity, and compressive mechanical strength. Also, hydrogels underwent biomineralization in simulated body fluid (SBF) for 14 days, forming bone-like apatite with a Ca/P ratio similar to hydroxyapatite [239].

Nanocellulose has been widely used in bone tissue engineering owing to its biocompatibility and reinforcing properties. However, its application presents challenges such as anisotropic microstructure and biomechanics. To address such challenges, swelling-induced nanofiber alignment and in situ biomineralization strategies were developed to prepare bacterial cellulose/cellulose nanofibrils scaffolds for bone marrow-derived mesenchymal stem cell differentiation. Electrostatic repulsion was induced by TEMPO-mediated oxidation paired with high-pressure homogenization, and tunable swelling was attained by incorporating urease into the network, which then facilitated the precipitation of CaCO_3_ crystals by immersion in a Ca^2+^ solution. Results showed hydrogels with tunable swelling anisotropic index and biomineralized scaffolds with higher mechanical strength efficiently supported cell growth and proliferation [244].

The formation of complex gradients is necessary for bone repair, prompting researchers to explore techniques for creating gradient scaffolds that imitate the native bone’s hierarchical structure. Zhang [245] and colleagues developed scaffolds using poly (vinyl alcohol)/bacterial cellulose with varying hydroxyapatite (HAp) content through a buoyancy-driven gradient (BG) approach. The gel scaffolds exhibited a microstructure with a gradient of hydroxyapatite, leading to improved adhesion, spreading, and proliferation of MC3T3-E1 cells and exceptional osteogenic capability. Furthermore, in subcutaneous implantation models in mice, these scaffolds displayed better biocompatibility than other scaffolds tested in vitro.

**Figure 11:**
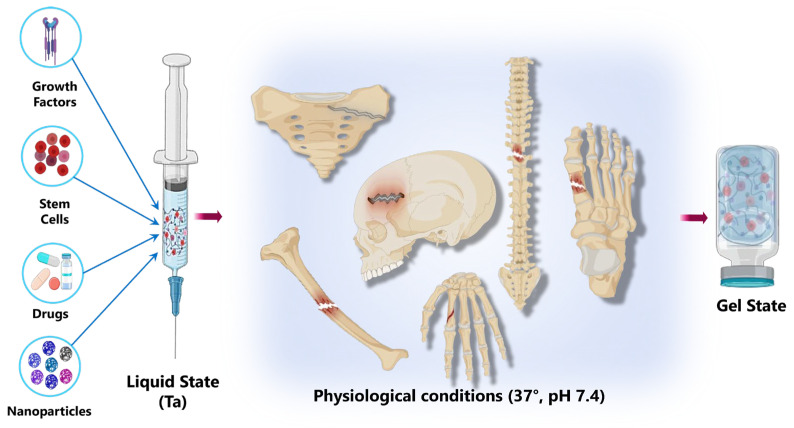
Cellulose-based injectable hydrogels loaded with growth factors, cells, drugs such as antibiotics or anti-inflammatory agents, and nanoparticles in a liquid state before physiological conditions and gel state after them.

In situ forming injectable hydrogels have recently been developed for minimally invasive bone repair. They are specifically designed to replicate the exact shape of the damaged area using conventional bone materials, ensuring seamless, durable, and customized bone reconstruction (See Figure 11). The materials in question can gel quickly in vivo, enabling them to fill irregular areas effectively. This property facilitates minimally invasive transplantation procedures while also allowing the transport and delivery of encapsulated cells, drugs, and bioactive molecules [246]. For instance, Qui et al. [238] developed an injectable, photocurable hydrogel using modified carboxymethyl cellulose and spherical hydroxyapatite to mimic natural bone matrix. This hydrogel promoted cell proliferation, supported adhesion, and the expression of osteogenic-related genes in MC3T3-E1 cells in vitro. Additionally, it enhanced the activity of critical proteins involved in bone formation, thus promoting bone regeneration in skull defects, and it showed good compatibility and stability when implanted subcutaneously. In a different example, Peyraviana et al. [247] have developed an injectable hydrogel comprising oxidized carboxymethyl cellulose/carboxymethyl chitosan with an angiogenesis stimulator peptide (QK) to promote angiogenesis effectively. This hydrogel exhibited positive properties such as swelling, water absorption, and degradation over 21 days, ensuring continual peptide release. Moreover, the hydrogel containing the QK peptide significantly enhanced the proliferation, differentiation, angiogenesis, and osteogenic potential of both Bone Marrow Mesenchymal Stem Cells (BM-MSC) and human umbilical vein endothelial cells (HUVEC). Notably, molecular and histological assessments confirmed increased expression of key genes, resulting in the successful inhibition of femoral head necrosis and a marked increase in blood vessel formation in the affected area.

Three-dimensional bioprinting is a promising method for creating scaffolds for bone tissue engineering. Since the complex structure of natural bone can be replicated using 3D-printing bioinks, these bioinks are typically loaded with biomolecules and cells to accelerate bone growth and tissue regeneration. However, there are limitations to incorporating cells into inks (i.e., enhancing cell viability, scaling up efficiently, promoting maturation, increasing architectural complexity, and ensuring adequate resolution), which affect the bioprinting process and the choice of biomaterials that can be used [248]. Afra et al. [249] developed bioinks for 3D printing personalized bone scaffolds to treat osteoporosis-induced bone defects. The bioinks included chitosan, nanohydroxyapatite, sodium alendronate, and hydroxyethyl-cellulose. The printed scaffolds were crosslinked using citric acid or KOH and coated with collagen and gelatin. They were compatible with mesenchymal stem cells and the MG-63 cell line and exhibited sustained release of alendronate over 50 days. This led to a significant decrease in Cathepsin K expression, indicating a potential to inhibit osteoclast activity. 

### 5.5. Skeletal Muscle Tissue Engineering

The functional abilities of skeletal muscle are intricately tied to its well-organized microstructure, primarily composed of parallel-oriented myotubes. Significant muscle loss impedes the body’s natural regeneration process, forming scar tissue that hampers the original muscle structure and functionality. To address this, efforts have been made to engineer skeletal muscle tissue using suitable biomaterials, cells, muscle progenitors, and precise 3D architectures to aid and accelerate regeneration [250]. In addition, tissue engineering designs scaffolds to precisely direct and regulate the behavior of cells toward specific lineages, strengthening the regenerative potential of transplanted cells [251]. For this purpose, Rzhepakovsky et al. [252] designed scaffolds based on bacterial cellulose and gelatin. The hydrogels significantly increased swelling, porosity, and structural stability compared to a bacterial cellulose scaffold. Additionally, the hydrogel displayed a remarkably high level of biocompatibility when tested in an artificial environment or in vivo by implanting the hydrogel onto the chicken embryo chorioallantoic membrane and subcutaneously implanting it in rats. The results revealed a highly organized formation of connective tissue structures and vascularization. In this same context, an innovative scaffold was created using gelatin, alginate, and ε-Poly-l-lysine hydrogel for 3D bioprinting of scaffolds to cultivate skeletal muscle cells. These 3D printed scaffolds featured a carefully designed porous structure that effectively supported attachment, proliferation, and differentiation of both porcine muscle stem cells (PMuSC) and C2C12 mouse skeletal myoblasts. Notably, these scaffolds demonstrated excellent biocompatibility, as evidenced by the high viability and expression of desmin in the extracted PMuSCs, indicating robust myogenesis [253].

External stimuli have been used to improve the performance of cellulose-based hydrogels, for instance, scaffolds with customizable physical and mechanical properties were developed using bacterial cellulose and were subsequently used for in vitro cultured human skeletal muscle myoblasts [254]. The study revealed that maturation indicators of human skeletal muscle myoblasts showed significant improvement in the 3D bacterial cellulose scaffold culture compared to the 2D reference. Furthermore, electrical impulse application enhanced cellular function, resulting in a highly aligned morphology of human skeletal muscle myofibers. Additionally, compared to commercially available scaffolds and a gelatin laboratory-made methacryloyl hydrogel, it was found that the bacterial cellulose scaffold outperformed them in terms of strength and in vitro continued support of human skeletal muscle myoblasts. In another study, drawing inspiration from the process of muscle cell growth resulting from mechanical training, a team of researchers successfully developed a hydrogel with remarkable electrical properties, closely mimicking skeletal muscles. The hydrogel composition involved using partially depolymerized lignin as an interfacial binding agent, cellulose nanofibrils for reinforcement, and silver ions as the conducting medium within a polyvinyl alcohol matrix. This composite hydrogel showed high toughness, exceptional anisotropic strength, and high conductivity. Cellulose nanofibrils were incorporated into the polyvinyl alcohol hydrogel by creating dual physical enhancement networks, simulating the effects of muscle strengthening through mechanical training techniques [255].

### 5.6. Soft Tissue Engineering

Cellulose-based hydrogels are a popular choice for tissue-engineered scaffolds because they have mechanical, biological, and physical properties similar to those of the extracellular matrix (ECM). The three-dimensional structure of hydrogels makes them an ideal matrix for the growth of smooth muscle cells (SMC), which are typically arranged in multiple layers within smooth muscle tissues [256]. For example, Khan et al. [257] have successfully created a bilayer material for soft tissue engineering. They crosslinked the top layer of poly(vinyl alcohol)/bacterial cellulose to an interconnected porous 3D gelatin/PVA hydrogel bottom layer. The top layer exhibited random cellulose deposition on the surface while aligned in the cross-section. Additionally, the composed hydrogel displayed a hydrophilic surface, a high degree of swelling, and good stability. Furthermore, the hydrogel could sustain and control the release of Ag-sulfadiazine and can be effective against *Escherichia coli*, *Staphylococcus aureus*, and *Pseudomonas aeruginosa*. While in vitro studies involving fibroblasts (3T3) and human embryonic kidneys (HEK-293) demonstrated desirable cell viability, proliferation, and adhesion to the bilayer.

Another study used the temperature-sensitive phase behavior of hydroxypropyl cellulose to fabricate hydrogels with interconnected macroporous structures in an aqueous setting. The hydroxypropyl cellulose (HPC) was subjected to modification using allyl isocyanate. Subsequently, the influence of temperature on their phase behavior was investigated, considering the degree of modification (DS). A derivative with a degree of modification of 1.5 was chosen for scaffold preparation. The aqueous solutions were heated to induce the development of biphasic systems. In this condition, they were immobilized effectively by crosslinking and exposure to gamma-ray irradiation. The crosslinked hydrogels were lyophilized, resulting in 3D macroporous sponges. The reconstituted gels exhibited an interconnected macroporosity, high water content, and robust mechanical stability towards soft tissues. The study successfully confirmed cytocompatibility across many cell types, and the in vivo biocompatibility test revealed a limited inflammatory response over a 12-week subcutaneous implantation in mice [258].

Cellulose-based hydrogels can also be designed to change their physical or mechanical properties in response to mechanical forces, mimicking the human body’s reactions. This includes responding to pressure, compression, and strain by adjusting the bonds and interactions within the hydrogels. These hydrogels can become stiffer when stretched, repair themselves, and relieve stress when strained. As such, researchers developed mechanically responsive carboxymethylcellulose hydrogels with pendant boronated esters crosslinked with tannic acid. They studied the properties of these materials in terms of temperature, polymer concentration, and crosslinking density. The hydrogels exhibited self-healing and strain memory, strain-dependent stress relaxation, and shear rate-dependent changes. These characteristics were attributed to the dynamic nature of the boronated ester and intermolecular interactions such as interchain hydrogen bonding and bundling [259].

Autologous platelet concentrates contain growth factors that promote cell migration, proliferation, differentiation, and angiogenesis and, therefore, tissue regeneration [260]. These concentrates can be encapsulated into hydrogels, thus creating bioinks suitable for 3D printing. In this regard, Grandjean et al. [261] developed a bioink made of alginate/cellulose hydrogel, loaded with thrombocyte concentrate, demonstrating excellent biocompatibility when tested with human osteogenic sarcoma cells. The printed constructs released pro-angiogenic growth factors and showed proper cell viability and proliferation when cultivated with primary human umbilical vein endothelial cells. Detailed proteome and secretome analysis in vivo revealed a significant presence of pro-angiogenic proteins in the 3D structure.

### 5.7. Nervous System Tissue Engineering

The nervous system is a sophisticated network that comprises the central and peripheral nervous systems, containing the brain, spinal cord, and peripheral nerves. It accurately processes sensory signals and transmits responses to targeted areas. In cases of injury due to trauma or disease, grafting becomes necessary for severe damage and notable brain injuries that can pose life-threatening risks. In this respect, tissue engineering has significantly advanced in developing nervous tissue constructs that seamlessly integrate with the body’s existing neural networks to facilitate and promote nerve regeneration. In this respect, essential neural tissue engineering tools are hydrogels. Cellulose hydrogels have the potential to closely resemble neural tissue in terms of composition and structure, making them suitable for restoring damage to the central and peripheral nervous systems and for delivering biological agents and drugs for nerve repair and regeneration. Additionally, their biocompatibility and ability to release functional ions make them able to replicate the biological and electrical properties of human neural tissue, making them effective for clinical translation in treating nervous system disorders [262]; for instance, a novel double network hydrogel was prepared by coupling a network of carboxymethyl cellulose-g-acrylic acid/iron oxide nanoparticles with a polyacrylamide network. The hydrogel structure demonstrated remarkable mechanical strength despite containing 90% water. Its impact on nerve cells was non-cytotoxic and did not disrupt or hinder their growth and proliferation [263]. Similarly, Najafi et al. [264] designed a hydrogel of carboxymethyl cellulose-starch/copper-doped carbon quantum dots to protect and control the release of quercetin. The hydrogel was meant to target malignant brain tumors and could load and encapsulate high amounts of the drug, among the top reported amounts for similar systems. In addition, it selectively displayed notable cytotoxicity, reducing the viability of U87-MG cancer cells while maintaining high cell viability of normal L929 healthy cells. Another study presented a conductive hydrogel that efficiently carried and released neurotransmitters such as dopamine in the brain of Parkinson’s disease patients. The hydrogel made of sodium carboxymethylcellulose-poly(3,4-ethylene dioxythiophene) microparticles could release a hundred percent of dopamine in five days using small electrical stimuli daily [265].

Recent studies have highlighted the potential of injectable hydrogels as biodegradable scaffolds for central nervous system tissue engineering. These biomimetic hydrogels closely resemble various aspects of the central nervous system, and they offer the advantage of minimal invasiveness when injected into target areas, along with fewer adverse secondary effects and lower costs. In addition, these hydrogels can promote faster regeneration, minimize pain, and enable easier use and implantation [266,267]. On this basis, Belyaeva et al. [268] developed a hydrogel for filling brain postoperative cavities while releasing antitumor drugs. The hydrogel based on nanocrystalline cellulose grafted with poly(N-isopropylacrylamide) remained liquid, keeping the injection conditions, and turned into a gel at physiological temperature after being placed in the targeted area. The hydrogel was discovered to have a fibrous structure and mechanical characteristics akin to brain tissue, such as non-linear mechanics like strain-stiffening and compression softening due to the rod-shaped nanocellulose present. Furthermore, the hydrogel was biocompatible with primary brain cells and showed antitumor activity through the controlled release of paclitaxel, an antitumor drug.

Spinal cord injury is a severe neurological condition with limited treatment options. It is characterized by quadriplegia, paraplegia, quadriparesis, and autonomic dysfunction [269]. In this respect, tissue engineering aims to solve this condition by mimicking spinal cord regeneration conditions. For this purpose, a bioactive hydrogel with a dual porous structure network prepared of self-assembly peptide nanofibers and natural cellulose nanofibers exhibited excellent affinity to neurons, biodegradability, biocompatibility, and self-healing ability, which promoted spinal cord repair and regeneration. The in vitro experiments demonstrated that the injected hydrogel facilitated the proliferation and migration of bone marrow mesenchymal stem cells while inducing neural stem cell proliferation, differentiation, and axonal growth. Additionally, it was observed that the hydrogel attenuated spinal cord post-inflammation inhibited reactive astrocyte proliferation and facilitated endogenous neuron proliferation and migration [270].

**Figure 12:**
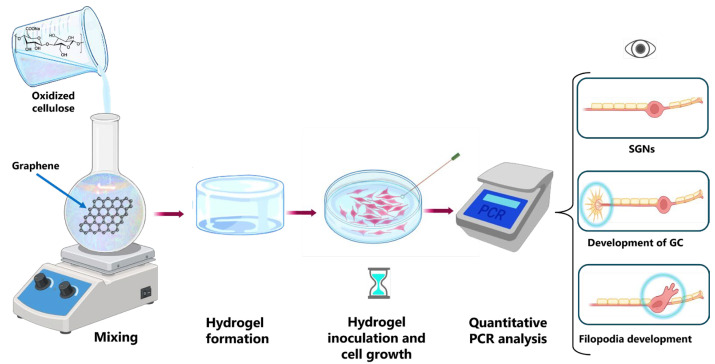
Bacterial cellulose–graphene hydrogel for culture and growth of spiral ganglion neurons (SGNs) and development of growth cones (GC) and filopodia.

Hearing is another function controlled by the neural system through spiral ganglion neurons, which relay acoustic information from sensory hair cells to the brain stem. When spiral ganglion neurons suffer damage, their inability to regenerate leads to hearing loss [271]. Shi et al. [272] tailored a bioactive hydrogel combining graphene oxide (GO) and TEMPO-oxidized bacterial cellulose to create a suitable microenvironment for the growth of spiral ganglion neurons. The hydrogel mimicked the structure and morphology of the extracellular matrix. Quantitative PCR results confirmed that the hydrogel could accelerate growth cones and filopodia development by increasing mRNA expression levels of diap3, fscn2, and integrin β1, which suggests the hydrogel could be used to construct biomimetic nerve grafts for nerve defect repair or replacement (see Figure 12) [272].

The eyes are an integral part of the central nervous system, and unfortunately, the eye nerves cannot regenerate. Therefore, researchers are exploring neural tissue engineering as a promising approach for treating eye nerve conditions. From this perspective, a recent study drew inspiration from the structure of collagen fibers in the corneal stroma to develop a novel hydrogel system. The system, created using a combination of copper-tannic acid nanozymes, hyaluronic acid, tannic acid, carboxymethyl cellulose, and methacrylate, displayed a directional arrangement at both micro and macro scales. The hydrogel exhibited significant antifungal properties, effectively inhibiting the growth of certain fungi. In vitro experiments showed that fibroblasts interacted with the hydrogel in a way that promoted cell proliferation and directional migration, leading to the alignment of collagen fibers and facilitating repair of the corneal stroma in vivo. Additionally, the study found that downregulating certain growth factors inhibited myofibroblast differentiation and promoted corneal healing [273].

Keratoplasty is a standard procedure used to treat severe corneal disorders. It involves replacing a damaged or diseased cornea with a healthy cornea from a donor to improve overall eye health. However, the outcome may be impacted by challenges such as the rejection of grafts, substantial expenses, and a shortage of donors. In this context, tissue engineering has utilized biodegradable polymers to create 3D-printed tissue replicas with the same intricate architecture [274]. For example, researchers developed 3D hydrogels for corneal stroma using a combination of methylcellulose and gelatin methacryloyl. By adjusting the pressure and print speed during the manufacturing process, the hydrogels achieved a level of transparency that closely resembled that of the natural cornea. Additionally, the in vitro studies using goat corneal stromal cells revealed that the hydrogel model effectively emulated the biophysical properties of the native corneal stroma. Furthermore, it proved a capacity to facilitate cell adhesion and proliferation [274].

### 5.8. Cardio and Vascular Tissue Engineering

The human heart is a crucial organ with a limited ability to heal after injury or disease. While heart transplantation is a life-saving option, it faces challenges such as donor shortages and potential immune reactions. Therefore, tissue engineering aims to create artificial heart tissue to aid in heart substitution or repair, making significant strides in creating complex three-dimensional spatial structures that mimic in vivo functions. This includes using cell sources, biomaterial-based scaffolds, and biochemical cues to create cardiac muscles, vascular substitutes, and heart valves [275]. Bioactive and biodegradable hydrogels are crucial in delivering cells to heart-damaged tissue by providing a hydrated and porous 3D structure. These hydrogels are essential for regenerating heart tissue and replicating natural cardiac tissue microenvironments. They also support myocardial wall stress and help preserve cells [276]. In this instance, Tohidi et al. [277] developed an injectable hydrogel with potential heart and nerve tissue engineering applications. The hydrogel was made from collagen, hyaluronic acid, 3-aminopropyl-triethoxysilane-g-oxidized bacterial cellulose, and gold nanoparticles. Also, the hydrogel demonstrated thermosensitivity and self-healing properties, and compared to the base matrix, it showed improved elasticity and a lower gelation temperature. When electrically stimulated, the hydrogel responded at physiological temperatures and showed low cytotoxicity after 72 h of incubation.

Mechanical and biological heart valve prostheses may develop several significant drawbacks over time despite their satisfactory short-term performance. Prostheses, made of various materials, may require long-term anticoagulation treatments and can lead to complications such as thromboembolism and endocarditis. Therefore, it is critical for the structural and functional characteristics of a scaffold to closely match those of the materials present in the extracellular matrix (ECM) [278]. In this regard, Ma et al. [279] engineered valve leaflets that exhibited unique elastic characteristics, promoted a stable fibroblast phenotype, and showed resistance to osteogenic differentiation by grafting TEMPO-modified nanocrystalline cellulose onto the backbone of methacrylate gelatin. The study investigated how human adipose-derived mesenchymal stem cells (HADMSC) behaved when encapsulated within the hydrogel and cultured in standard and osteogenic media for 14 days. The results showed that compared to the control group, the HADMSC encapsulated within the hydrogel decreased alpha-smooth muscle actin expression and increased expression of vimentin and aggrecan, suggesting a quiescent fibroblastic phenotype. Moreover, the encapsulated cells showed lower osteogenic gene expression under osteogenic conditions, indicating potential calcification resistance. Furthermore, the material was successfully used for 3D bioprinting a self-standing tubular structure that sustained cell viability [279].

Extracellular vesicles (EVs) have been recognized as one of the promising specific treatments for myocardial infarction prognosis. A silk fibroin/hydroxypropyl cellulose composite hydrogel was engineered with AC16 cell-derived targeted modification; folic acid-functionalized EVs were modified with distearoylphosphatidyl ethanolamine–polyethylene glycol (DSPE-PEG-FA) through noncovalent interactions to target and accelerate myocardial infarction repair. In vitro, cytocompatibility analyses revealed that the prepared hydrogels had excellent cell viability, and the functionalized EVs displayed higher cell migration in scratch assays. In vivo, the composite hydrogels promoted myocardial tissue repair in a rat model by delaying the progression of myocardial fibrosis and enhancing angiogenesis in the infarct area [280]. 

## 6. Conclusions

Cellulose and its derivatives represent a remarkable class of sustainable biomaterials that exhibit unique advantageous properties for applications in tissue engineering. These biopolymers are characterized by their exceptional capacity to form hydrogels, which can mimic the natural extracellular matrix, providing an ideal environment for cell growth and development. Furthermore, cellulose-based hydrogels have gained significant attention due to their biocompatibility, biodegradability, hydrophilicity, and controllable structure. This paper reviewed the structure and properties of cellulose and its derivatives from different sources. Additionally, physical and chemical crosslinking techniques for synthesizing hydrogels and the mechanisms and requirements for hydrogel formation were discussed. The discussion also encompassed up-to-date technologies related to the fabrication and potential applications of cellulose-based hydrogels tailored for various anatomical structures in the body. However, several challenges that hinder the optimal application of cellulose-based hydrogels in tissue engineering still need to be addressed. For instance, the difference between the pore size of a hydrogel and the size of living cells is an issue that may hinder cell growth. Moreover, attention must be placed on tuning suitable biomechanical properties when designing three-dimensional structures, as incorrect mechanical modulus results in unstable matrices. Also, an adequate level of cell adhesion is necessary to prevent cells from being repelled and to promote attachment and growth within the hydrogel matrix. Another important aspect is enhancing the biological characteristics that favor cell interaction and tissue regeneration. This involves meticulously selecting suitable cell types that align with the intended tissue type. Additionally, incorporating growth factors can significantly influence cell proliferation, differentiation, and angiogenesis. Including therapeutic drugs can be pivotal in modulating inflammatory responses and promoting healing processes. Also, the presence of bioactive cues plays a critical role in guiding cellular behaviors. By integrating these elements thoughtfully, hydrogels can support cellular functions and foster the complex processes involved in tissue regeneration. In conclusion, upcoming research will focus on designing and tailoring hydrogels for specific applications. This includes exploring safer and more sustainable materials and synthesis techniques, identifying less invasive medical treatment methods, and translating these studies into practical applications.

## Figures and Tables

**Table 1:** Cellulose polymorphs characteristics.

Unit	Occurrence	Precursor	Structure	Arrangement	Reference
I_α_	Natural: bacteria, alga, tunicates	Glucose	Triclinic	One chain (42 atoms) per unit, parallel	[1,2,3,4]
I_β_	Natural: plants	Glucose	Monoclinic	Two parallel chains per unit (84 atoms)	[1,2,3,4]
II	Synthetic: (a) chemical regeneration, (b) caustic mercerization, (c) alkaline salt precipitation, and (d) microbiological cultures	Cellulose I	Monoclinic	Two antiparallel chains per unit	[1,2,3,4]
III_I_	Synthetic: (a) exposure to amines or ammonia at 140 °C, (b) removal by evaporation	Cellulose I_β_	Monoclinic	One cellulose chain per unit, parallel	[4,8,9,10,11,12]
III_II_	Synthetic: (a) exposure to amines or ammonia at 140 °C, (b) removal by evaporation	Cellulose II	Monoclinic	Undefined	[4,8,9,10,11,12]
IV_I_	Natural and synthetic: (a) glycerol heat treatment at 260 °C, (b) super-critical ammonia treatment at 105 °C	Cellulose III_I_	Orthorhombic	Two antiparallel chains per unit	[8,13,14,15]
IV_II_	Synthetic: (a) glycerol heat treatment, (b) deacetylation at 150–160 °C	(a) Cellulose III_I_ and III_II_(b) Cellulose triacetate	Undefined	Two parallel chains per unit	[13,14,15,16,17,18]

**Table 2:** Cellulose, hemicellulose, and lignin content of some plant fibers.

Source	Cellulose%	Hemicellulose%	Lignin%	Reference
Cotton	82–96.4	2–6	0–5	[32]
Cotton stalks	5%	20%	21	[33]
Birch	40.0	36	20	[34]
Beech	41	33	22	[34]
European aspen	60.0	15	12	[35]
Nerium oleander	45	15	21	[35]
Asclepias	43.8	16	8.6	[35]
Flax	63–71	12–21	2–3	[36]
Hemp	63–64	12–15	3–6	[37]
Jute	64.4	12	11.8	[38]
Kenaf	49.88	13.82	10.33	[39]
Sisal	50–74	10–14	8–11	[40]
Ramie	68–76	13–15	0.6–1	[32]
Coir	32–43	10–20	43–49	[36]
Bamboo	73.8	12.5	10.1	[41]
Rice straw	28–45	12–32	5–24	[42]

**Table 3:** Hydrogel responses to different stimuli.

Response	Transition		
Size change (swelling–deswelling)	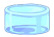 Hydrogel	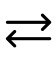	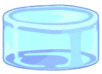
	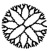 Dendrimer hydrogel	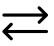	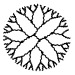
	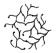 Hyperbranched hydrogel	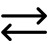	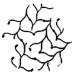
Morphology change	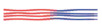 Block copolymer	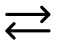	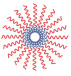 Micelle
	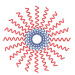	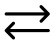	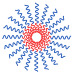 Reverse micelle
	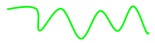 Coiled	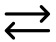	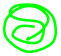 Globular
	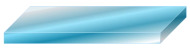 Planar	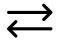	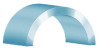 Curved
	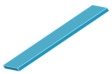 Stripe	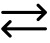	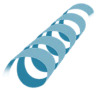 Coiled
Degradation	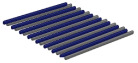 Sintered	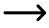	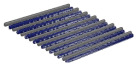 Degraded
Phase transition	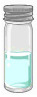 Liquid	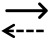	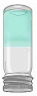 Gel

**Table 4:** pH-responsive polymers and pH-cleavable linkers.

pH-Responsive Cationic Polymers	pH-Responsive Anionic Polymers	pH-Cleavable Linkers
Poly(2-dimethylaminoethyl methacrylate)	Poly(acrylic acid)	Ortho-esters
Poly(2-diethylaminoethyl methacrylate)	Poly(2-carboxyethyl acrylate)	Ketals/acetals
Poly(2-diisopropylaminoethyl methacrylate)	Poly(2-propylacrylic acid)	Hydrazone
Poly(4-vinylpyridine)	Poly(aspartic acid)	Imines
Poly(4-(1H-imidazol-1-yl)butyl methacrylate)	Poly(4-vinylbenzoic acid)	Maleic acid
Poly(lysine)	Poly(glutamic acid)	Amide derivatives
Poly(histidine) (PHis)	Poly(vinylsulfonic acid)	Silyl ethers
Poly(ethylenimine) (PEI),	Poly(vinylphenylboronic acid)	Trityl derivatives
Poly(β-amino ester) (PbAE).	Poly(vinylphenylboronic acid)	
Chitosan	Hyaluronic acid	
Dextran	Sodium alginate	
	Xanthan	
	Carragenan	
	Chondroitin sulfate	
	Fucan and flucoidans	
	Mannan and galactomannan	

**Table 5:** Polymers exhibiting LCST and UCST behavior.

LCST Behavior	UCST Behavior
Poly (N-isopropylacrylamide)	Poly(N-acryloyl glycinamide)
Poly(N-vinyl caprolactam)	Poly(sulfopropyl dimethylammonium propylacrylamide)
Poly(N,N-diethylaminoethyl methacrylate)	Poly (2-(N-3-Sulfopropyl-N,N-dimethyl ammonium)ethyl methacrylate)
Poly(2-(N-morpholine) ethyl methacrylate)	Polymethacrylamide
Poly(oligo(ethylene glycol)methacrylate)	
Poly(N,N-diethylacrylamide)	
Poly(N,N-dimethylaminoethyl methacry-late, poly(2(diethylamino)ethyl acrylamide))	

## Data Availability

No new data were created or analyzed in this study. Data sharing does not apply to this article.
